# Quantitative Profiling of Hydroxy Lipid Metabolites in Mouse Organs Reveals Distinct Lipidomic Profiles and Modifications Due to Elevated n-3 Fatty Acid Levels

**DOI:** 10.3390/biology6010009

**Published:** 2017-02-04

**Authors:** Cheng-Ying Chiu, Christopher Smyl, Inci Dogan, Michael Rothe, Karsten-H. Weylandt

**Affiliations:** 1 Department of Medicine, Division of Hepatology and Gastroenterology, Charité University Medicine Berlin, Campus Virchow-Klinikum, 13353 Berlin, Germany; Cheng-Ying.Chiu@charite.de (C.-Y.C.); christopher.smyl@charite.de (C.S.); 2 Lipid Clinic, Experimental and Clinical Research Centre (ECRC), Charité University Medicine and Max-Delbrück-Center for Molecular Medicine, 13125 Berlin, Germany; 3 Lipidomix, 13125 Berlin, Germany; inci.dogan@lipidomix.de (I.D.); michael.rothe@lipidomix.de (M.R.)

**Keywords:** arachidonic acid, eicosapentaenoic acid, docosahexaenoic acid, HETE, HEPE, HDHA, fat-1, polyunsaturated fatty acids, omega-6, omega-3

## Abstract

Polyunsaturated fatty acids (PUFA) are precursors of bioactive metabolites and mediators. In this study, the profile of hydroxyeicosatetraenoic (HETE), hydroxyeicosapentaenoic (HEPE) and hydroxydocosahexaenoic (HDHA) acids derived from arachidonic acid (AA), eicosapentaenoic acid (EPA) and docosahexaenoic acid (DHA) in colon, liver, lung, spleen, muscle, heart and kidney tissue of healthy wildtype mice were characterized, and compared to profiles in organs from transgenic fat-1 mice engineered to express the *Caenorhabditis elegans* fat-1 gene encoding an n-3 desaturase and thereby with endogenously elevated n-3 PUFA levels. PUFAs were measured using gas chromatography. The lipid metabolites were assayed using LC-MS/MS. AA and DHA were the prominent PUFAs in wildtype and fat-1 mice. EPA levels were low in both groups even though there was a significant increase in fat-1 organs with an up to 12-fold increase in fat-1 spleen and kidney. DHA levels increased by approximately 1.5-fold in fat-1 as compared to wildtype mice. While HETEs remained the same or decreased moderately and HDHAs increased 1- to 3-fold, HEPE formation in fat-1 tissues increased from 8- (muscle) to 44-fold (spleen). These findings indicate distinct profiles of monohydroxy lipid metabolites in different organs and strong utilization of EPA for HEPE formation, by which moderate EPA supplementation might trigger formation of biologically active EPA-derived resolvins.

## 1. Introduction

Long-chain polyunsaturated fatty acids (PUFA) such as the essential omega-3 and omega-6 polyunsaturated fatty acids (n-3 and n-6 PUFA) are involved in a wide range of biological processes [1].

Beneficial effects of n-3 PUFA, mainly eicosapentaenoic acid (EPA, 20:5n-3) and docosahexaenoic acid (DHA, 22:6n-3), have been described in many animal models of diseases amongst others colitis, hepatitis, diabetes and cancer [2,3,4,5,6]. However, existing clinical data are conflicting, leading to uncertainty regarding the anti-inflammatory and anti-proliferative effect of n-3 PUFA [7,8,9,10]. The molecular mechanisms are only partially understood and include changes in membrane structures and gene expression as well as direct interactions with ion channels and due to their function as precursors of bioactive lipid mediators and metabolites [11].

The n-3 PUFAs compete with the predominant n-6 PUFA arachidonic acid (AA) as substrates of cyclooxygenase (COX), lipoxygenase (LOX), as well as cytochrome P450 enzymes (CYP 450) and therefore modulate the production and bioactivity of lipid mediators and metabolites. Not only are the pro-inflammatory AA-derived prostaglandins, leukotrienes, thromboxanes and hydroxyeicosatetraenoic acids (HETE) partially replaced by their less potent n-3 counterparts [12]; moreover hydroxylation of EPA and DHA can lead to hydroxyeicosapentaenoic acids (HEPE) and hydroxydocosahexaenoic acids (HDHA) respectively, of which a few, namely 18-HEPE, 17-HDHA and 14-HDHA, are precursors of highly anti-inflammatory and pro-resolving resolvins, protectins and maresins [13,14].

Recent advances in the field of lipidomics have enabled a detailed investigation of lipid product profiles [15,16,17]. A study demonstrated significant changes in the formation of LOX-derived 5-HETE, 12-HETE and 15-HETE with an association of higher levels and worse post-transplant graft function outcome in human kidney transplantation, indicating a critical role of lipid metabolites in the pathophysiology of post-transplantation organ dysfunction [18]. This is further supported by another study that found significantly elevated 5-HETE, 11-HETE, 12-HETE and 15-HETE levels in colonic mucosal biopsies from ulcerative colitis patients correlating with inflammation severity [19].

A previous study with transgenic fat-1 mice, engineered to express the *Caenorhabditis elegans* fat-1 gene encoding an n-3 desaturase and thereby with an abundance of n-3 PUFA in their organs and tissues [20], revealed increased levels of 18-HEPE and 17-HDHA in ionophore-activated blood [21]. This could be an indication for the formation of their anti-inflammatory and pro-resolving downstream pathway products from n-3 PUFA. Differences in the body levels of n-3 and n-6 PUFA could thus be an important predictor of lipid mediator and metabolite formation. However, to what extent the alteration of lipid product formation can indeed occur is unknown and may depend on both the availability of the parent fatty acid and the utilization of different PUFAs in a given tissue.

In this study, we set out to characterize the monohydroxy lipid metabolite profile in various organs of wildtype C57BL/6J mice. We then examined the effect of endogenously elevated n-3 PUFA levels on lipid metabolite formation. By using the transgenic fat-1 mouse model we were able to evaluate those effects without confounding factors that would typically arise from different diets. We found distinct characteristic lipid metabolite patterns in different organs as well as increased formation of n-3 PUFA derived metabolites in organs from fat-1 mice. Our findings further indicate highly efficient utilization of EPA for the formation of monohydroxy metabolites.

## 2. Materials and Methods

### 2.1. Lipid Compounds 

AA, EPA and DHA were purchased from Nu-Chek Prep (Elysian, MN, USA). Lipid mediators and the deuterated standard used in this study were purchased from Cayman Chemical (Ann Arbor, MI, USA). Substances used in this study were 4-Hydroxydocosahexaenoic acid (HDHA), 7-HDHA, 8-HDHA, 10-HDHA, 11-HDHA, 13-HDHA, 14-HDHA, 16-HDHA, 17-HDHA, 20-HDHA, 10,17-Dihydroxydocosahexaenoic acid (DiHDHA), 5-Hydroxyeicosapentaenoic acid (HEPE), 8-HEPE, 9-HEPE, 12-HEPE, 15-HEPE, 18-HEPE, 5-Hydroxyeicosatetraenoic acid (HETE), 8-HETE, 9-HETE, 11-HETE, 12-HETE, 15-HETE, Resolvin D1 (RvD1), 15-HETE-d8.

### 2.2. Animals and Sample Collection

Transgenic fat-1 C57BL/6J mice were generated as previously described [20]. Mice were phenotyped according to the n-6:n-3 PUFA ratio in their tails as determined by gas chromatography. Heterozygous fat-1 male mice (*n* = 4) as well as their wildtype male littermates (*n* = 7) were group housed in pathogen free conditions at 12 h light/dark cycle and were fed a defined diet (Diet SM R/M-H + 10% safflower oil; ssniff Spezialdiäten GmbH, Soest, Germany). After 6 weeks on the diet all mice were anesthetized with isoflurane and sacrificed by cardiopuncture. All samples were collected and immediately snap frozen in liquid nitrogen and stored at −80 °C for further analysis. All animals received care according to the institutional guidelines of Charité Berlin and all efforts possible were made to minimize animal suffering. All experiments were carried out in accordance with EU Directive 2010/63/EU and use of the animals was registered and approved by the Landesamt für Gesundheit und Soziales Berlin (Reg No.: T0025/13).

### 2.3. Fatty Acid Analysis

#### 2.3.1. Sample Preparation for the Analysis of Fatty Acids

For PUFA analysis, approximately 100 mg tissue samples were homogenized in liquid nitrogen using a biopulverizer. Total fatty acids were extracted using a modified Morisson and Smith procedure as previously described [22,23], allowing for direct one-step transesterification of total fatty acids in tissue homogenates: An aliquot of each tissue homogenate was subjected to fatty acid methylation with 500 μL n-hexane (Carl Roth, Karlsruhe, Germany), 500 μL 12% boron trifluoride-methanol solution (Sigma-Aldrich, Steinheim, Germany) and 50 μL pentadecanoic acid (1 mg/mL; Sigma-Aldrich) as internal standard and incubated for 60 min at 100 °C. After cooling down to room temperature, fatty acid methyl esters were extracted in the hexane phase following the addition of 750 μL H_2_O. The samples were vortexed for 4 min and centrifuged (5 min, 3500 rpm). The upper hexane phase was removed and stored at −20°C until analysis.

#### 2.3.2. Gas Chromatography (GC)

Fatty acid methyl esters were analyzed by GC using a HP 5890 GC System (Agilent, Santa Clara, CA, USA) equipped with a flame-ionization detector. The chromatography utilized an Omegawax capillary column (L × I.D. 30 m × 0.25 mm; Sigma-Aldrich). Fatty acids were identified by matching their GC retention time with those in a PUFA standard (Nu-Chek-Prep) and further analyzed using GC ChemStation Software (Agilent). The concentration of fatty acids was calculated using the internal standard curve in relation to the weighed-in quantity. Standard curves of the assayed fatty acids are provided in the supplements (Figure S1).

### 2.4. Lipid Metabolite Analysis

#### 2.4.1. Sample Preparation for the Analysis of Lipid Metabolites

Analysis was performed essentially as described before [16]. Materials used for alkaline hydrolysis and solid phase extraction (SPE) were obtained from Carl Roth, Karlsruhe, Germany. Methanol was purchased from Merck, Darmstadt, Germany. Briefly, 30 mg tissue samples were mixed with 0.5 mL distilled water, 0.5 mL methanol containing 0.1% butylated hydroxytoluene, and 0.1 mL internal standard containing 10 ng 15-HETE-d8. The samples were then hydrolyzed after adding 300 μL sodium hydroxide 10 M for 30 min at 60 °C. Control experiments using pure fatty acid solutions of AA, EPA or DHA or spiked plasma samples demonstrated that the hydrolysis step did not increase autooxidation, confirming the validity of this analysis approach that was also used in previous studies [24,25].

Then the samples were brought to pH 6 with 10 M acetic acid 1 M sodium acetate buffer. An aliquot of 50 μL was taken for measurement of total proteins using a Modified Lowry protein assay kit (Thermo Scientific Pierce, Loughborough, UK). After centrifugation, the obtained supernatant was added to Bond Elute Certify II columns (Varian, Palo Alto, CA, USA) for SPE. Columns were preconditioned with 3 mL methanol, followed by 3 mL of 0.1 mol/L sodium acetate buffer containing 5% methanol (pH 6). The SPE-columns were then washed with 3 mL methanol/H_2_O (50/50 v/v). For elution 2 mL of n-hexane:ethyl acetate 25:75 with 1% acetic acid was used. The extraction was performed with a Visiprep SPE Vacuum Manifold (Sigma-Aldrich). The eluate was evaporated on a heating block at 40 °C under a stream of nitrogen to obtain a solid residue and stored at −20 °C until LC/ESI-MS/MS analysis was performed.

#### 2.4.2. LC/ESI-MS/MS

The residues were dissolved in 70 μL acetonitrile (Fisher Scientific, Loughborough, UK). Analysis was performed using an Agilent 1200 HPLC system with binary pump, autosampler and column thermostat (Agilent) with a Kinetex C-18, 2.1 × 150 mm, 2.6 μm column (Phenomenex, Aschaffenburg, Germany) using a solvent system of aqueous formic acid (0.1%) and acetonitrile (Fisher Scientific). The elution gradient was started with 5% acetonitrile, which was increased within 0.5 min to 55%, 14.5 min to 69%, 14.6 min to 95% and held there for 5.4 min. The flow rate was set at 0.3 mL/min, the injection volume was 7.5 μL. The HPLC was coupled with an Agilent 6460 Triplequad mass spectrometer with electrospray ionisation source (Agilent). The source parameters were as follows: Drying gas: 250 °C/10 L/min, Sheath gas: 400 °C/10 L/min, Capillary voltage: 4500 V, Nozzel voltage: 1500 V and Nebulizer pressure: 30 psi. Analysis was performed with Multiple Reaction Monitoring in negative mode. For details see Table S1. The concentration of metabolites was calculated using an internal standard calibration curve in relation to amount of total protein or the weighed-in quantity. Standard curves and response factors are provided in the supplements (Figure S2).

### 2.5. Statistical Analysis

Lipid metabolite and fatty acid levels were normalized to the weighed-in quantity of tissue sample. Statistical analysis was performed with Prism 5 Software (GraphPad, La Jolla, CA, USA). Comparison was made using the student’s *t*-test. All values are presented as the mean ± SEM or as indicated. Statistical significance was set at *p* < 0.05.

## 3. Results

**Figure 1:**
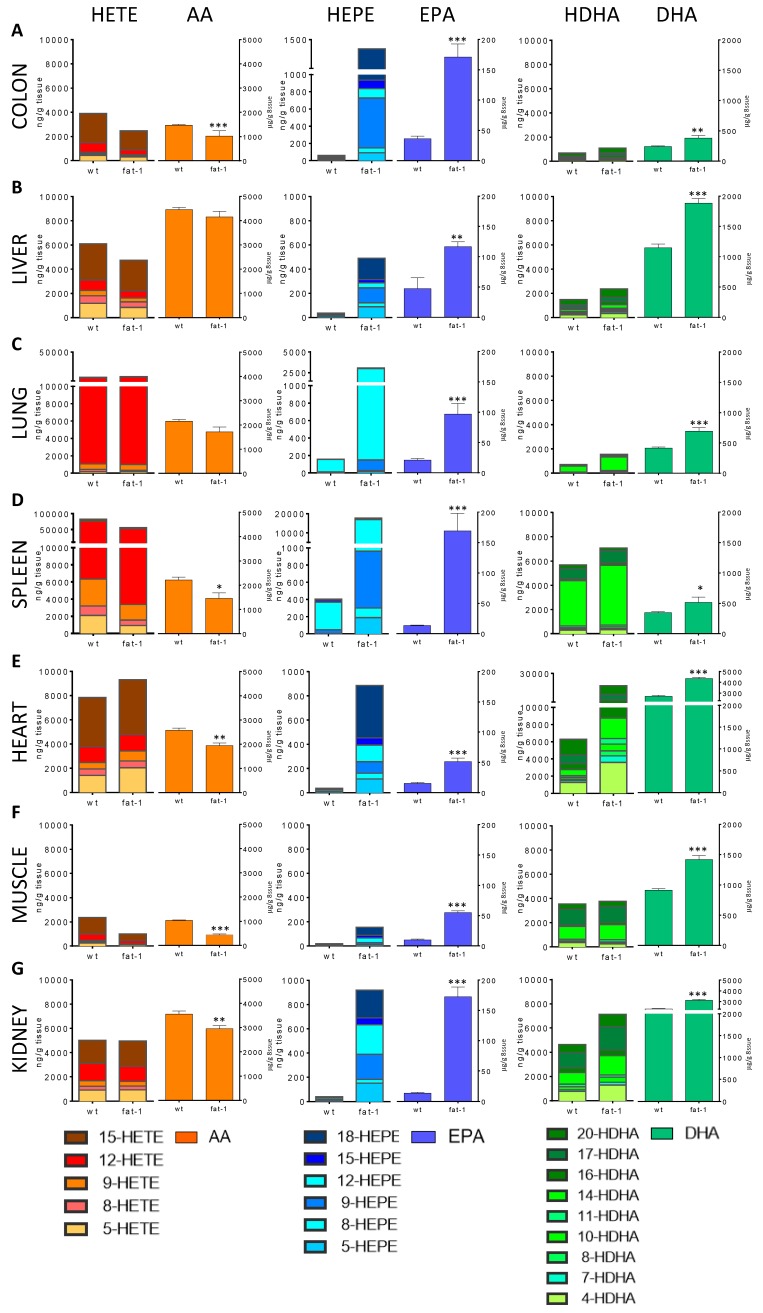
PUFA and lipid metabolite profile in organs of wildtype and fat-1 mice. Lipid metabolites and their precursor PUFAs (HETE and AA—left column; HEPE and EPA—middle column; HDHA and DHA—right column) in colon (**A**); liver (**B**); lung (**C**); spleen (**D**); heart (**E**); skeletal muscle (**F**) and kidney (**G**) from wildtype and fat-1 mice. Significant differences in PUFA levels between wildtype and fat-1 mice were shown as * *p* < 0.05, ** *p* < 0.01 and *** *p* < 0.001. The *p*-values for the differences of individual monohydroxy lipid metabolites in panels (**A**–**G**) are presented in the supplementary material (Tables S2–S8).

Total levels of the three PUFAs AA, EPA and DHA ranged between 1745 μg/g to 5873 μg/g in wildtype mice (Table 1). The lowest total PUFA levels were documented in colon and muscle tissue, while the highest total PUFA levels were found in heart, liver and kidneys. Relative distribution (in %) of AA:EPA:DHA in the colon (84:2:14, Figure 1A), spleen (85:1:14, Figure 1D), lung (83:1:16, Figure 1C), liver (79:1:20, Figure 1B) and kidney (61:0:39, Figure 1G) demonstrate the predominance of AA in most assayed organs. DHA was present in almost equal or higher amounts than AA in the muscle (53:1:47, Figure 1F) and heart tissue (49:0:51, Figure 1E). EPA was measured at low levels ranging from approximately 10–50 μg/g in wildtype tissues (Figure 1, right side of middle column).

Tissues of transgenic fat-1 mice revealed a partial and tissue-specific replacement of AA by EPA and DHA. AA levels were significantly lower as compared to their wildtype littermates in all organs except the liver and lung, where lower AA levels were also detected but failed to reach statistical significance (Table 1). However, AA remained predominant in the lung (68:4:28), spleen (67:8:25), liver (67:2:31) and colon (65:11:24) (Figure 1, left column). DHA levels were significantly higher in all assayed fat-1 organs; it was the most prevalent PUFA in kidney (47:3:50), heart (30:1:69) and muscle tissue (23:3:74) of fat-1 mice (Figure 1, right column). EPA levels, while still low, were also significantly higher and ranged between 50–170 μg/g (Figure 1, middle column).

Total monohydroxy lipid metabolite levels (HETEs, HEPEs and HDHAs) of more than 10,000 ng/g were detected in kidney, heart and lung of wildtype mice, while the total levels were nearly an order of magnitude higher in the spleen. Similar to their total PUFA levels, total monohydroxy lipid metabolite levels in colon and muscle tissue were the lowest. Interestingly, lipid metabolite levels were amongst the lowest in the liver tissue, which was rich in PUFA (Table 2).

Formation of monohydroxy lipid metabolites increased in the heart, kidney and lung of fat-1 mice, while monohydroxy lipid metabolite levels in the muscle tissue and spleen decreased. The total levels at nearly 84,000 ng/g in the spleen however remained the highest detected of all fat-1 tissues. Total levels in the colon and liver tissue remained constant compared to their wildtype littermates (Table 2).

AA-derived HETEs made up the biggest relative content of the assayed monohydroxy lipid metabolites in all tissues analyzed from wildtype mice, except for the muscle tissue, where DHA-derived HDHAs were predominant. Total HETE levels ranged between nearly 2500 ng/g in the muscle tissue to 86,500 ng/g in the spleen (Figure 1, left column). EPA-derived HEPEs were detected at very low levels reaching total HEPE levels between 20–60 ng/g in most assayed tissues. Higher levels were detected in the lung (156 ng/g) and spleen (403 ng/g) (Figure 1, middle column). In contrast, HDHAs were present in higher amounts in wildtype mice ranging from 740 ng/g in the lung and colon to nearly 6650 ng/g in the heart (Figure 1, right column).

Significantly lower total HETE levels were found in the colon, spleen and muscle tissue of fat-1 mice. Total HETE levels also decreased in the liver and kidney compared to wildtype mice, while higher levels—as opposed to the decrease of their parent fatty acid AA- were detected in the heart and lung tissue. However, those differences between these wildtype and fat-1 tissues failed to reach statistical significance. HETEs remained the predominant monohydroxy lipid metabolites in all organs, except for the kidney, muscle and heart tissue, where HDHA was prevalent. Total HDHA levels increased in all assayed organs. Similar to the fold differences of their parent fatty acid DHA, HDHA level fold differences ranged between 1.1- to 2.2-fold with the exception of the heart tissue where a significant increase by 3.4-fold was observed. HEPEs, in contrast, significantly increased in fat-1 mice with fold differences ranging between 8.1-fold in the muscle (157 ng/g) and 43.9-fold in the spleen (17,711 ng/g). While HDHAs were the predominant n-3 PUFA-derived lipid metabolites in wildtype mice, total HEPE levels exceeded total HDHA levels in the colon, lung and spleen of fat-1 mice (Table 2).

The colonic lipid metabolite patterns in wildtype and fat-1 mice were dominated by HETEs with a relative distribution of HETE:HEPE:HDHA 84:1:15 and 52:26:22 respectively (Table S2). 15-HETE was the main monohydroxy lipid metabolite followed by 12-HETE. Lower levels of all assayed HETEs were detected in the fat-1 mice, but only 15-HETE and 12-HETE were statistically significant (Figure 1A, left). HDHAs made up 15% and 22% of total monohydroxy lipid metabolites in wildtype and fat-1 mice respectively with a prevalence of 20-HDHA, 17-HDHA and 16-HDHA. All assayed HDHAs increased significantly in fat-1 mice with the exception of 8-HDHA (Figure 1A, right). EPA-derived HEPEs were detected at low levels in the wildtype colon and showed a marked increase in fat-1 mice, in particular 9-HEPE and 18-HEPE, overtaking HDHAs as the predominant n-3 PUFA-derived lipid metabolites (Figure 1A, middle).

The distribution of monohydroxy lipid metabolites in the liver of wildtype mice (80:0:20) and their fat-1 littermates (62:6:32) showed the predominance of HETEs (Table S3), mainly 15-HETE and 5-HETE (Figure 1B, left). HDHAs made up 20% and 32% of monohydroxy lipid metabolites in wildtype and fat-1 mice respectively, of which 20-HDHA, 17-HDHA and 4-HDHA were the most prominent (Figure 1B, right). While most HETEs decreased slightly in fat-1 mice, all assayed HDHAs increased significantly except for 4-HDHA and 11-HDHA. HEPEs increased significantly in fat-1 mice, with the main HEPEs being 18-HEPE and 9-HEPE (Figure 1B, middle).

HETEs dominated the lipid metabolite pattern of the lung tissue with HETE:HEPE:HDHA of 95:1:4 and 79:14:7 in wildtype and fat-1 mice respectively (Table S4). 12-HETE was by far the most prevalent lipid metabolite, followed by 11-HETE and 15-HETE. Low levels of HDHAs, mainly 14-HDHA, were found in the wildtype mice. Interestingly, 12-HEPE was also detectable. All assayed HEPEs and HDHAs, except for 7-HDHA, increased significantly in fat-1 mice with 12-HEPE becoming the second most prominent lipid metabolite (after 12-HETE) followed by 14-HDHA (Figure 1C).

Analysis of the spleen showed the highest tissue levels of monohydroxy lipid metabolites with relative distributions of HETE:HEPE:HDHA of 93:1:6 and 70:21:9 in wildtype and fat-1 mice respectively (Table S5). 12-HETE was the main metabolite; however, the other HETEs were also found in higher quantities than in the other assayed organs and tissues. The levels of HDHAs in the spleen of wildtype and fat-1 mice were similar with a predominance of 14-HDHA and 17-HDHA. HEPE levels increased from around 1% in wildtype mice to 21% of total monohydroxy lipid metabolites in the fat-1 mice. While 12-HEPE was already detected in prominent amounts in the wildtype mice, it showed a dramatic 49.4-fold increase in their fat-1 littermates (Figure 1D).

The lipid metabolite pattern in the heart tissue of wildtype (55:0:45) and fat-1 mice (29:3:68) mainly constituted of HETEs and HDHAs (Table S6). The predominant lipid metabolites were 15-HETE, 5-HETE, 12-HETE as well as 20-HDHA, 4-HDHA and 17-HDHA. Monohydroxy lipid metabolite formation in the fat-1 heart increased with higher levels of HEPEs, HDHAs and—in contrast to most of the other organs of fat-1 mice—HETEs. Furthermore, individual HDHAs increased by 2.8- to 4.4-fold to make up 68% of total monohydroxy lipid metabolites in the heart tissue, diverging from the rather strict regulation of HDHA formation in the other fat-1 tissues. HEPEs were present at low levels with a predominance of 18-HEPE in fat-1 mice (Figure 1E).

Low fatty acid levels led to low monohydroxy lipid metabolite levels in the muscle tissue. Consistent with the high content of DHA in muscle tissue, HDHAs were the predominant monohydroxy lipid metabolites with relative HETE:HEPE:HDHA distributions of 40:0:60 and 20:3:77 in the wildtype and fat-1 muscle tissue respectively (Table S7). 17-HDHA, 14-HDHA and 15-HETE were the major monohydroxy lipid metabolites, while HEPEs were found only at very low levels (Figure 1F).

Total HDHA levels were similar to the total HETE levels in the kidneys, as indicated by the relative distribution of HETE:HEPE:HDHA in the wildtype (52:0:48) and fat-1 mice (38:6:56) (Table S8). 15-HETE was the most prevalent HETE, followed by 12-HETE and 5-HETE in the kidneys. All HDHAs except for 11-HDHA increased in fat-1 mice with a predominance of 17-HDHA, 14-HDHA and 4-HDHA, but failed to reach statistical significance. Most HEPEs, while still low, increased significantly in fat-1 mice, with dominant amounts of 18-HEPE, 12-HEPE and 9-HEPE (Figure 1G).

## 4. Discussion

AA as well as EPA and DHA are synthesized through an alternating series of desaturations and elongations from linoleic acid (18:2n-6) and α-linolenic acid (18:3n-3). Since mammals lack the enzymes for the synthesis of the precursors as well as for interconversion between n-6 and n-3 PUFA, the polyunsaturated fatty acids are considered essential in the human diet [26].

Heterozygous fat-1 transgenic mice encoding an n-3 fatty acid desaturase from *C. elegans* have become an important tool in the study of omega-3 polyunsaturated fatty acids and have been used in the context of various murine disease models since their first description in 2004 [20]. As we were primarily interested in the effect of PUFA phenotype, heterozygous fat-1 mice were used also in this study in order to allow for a direct comparison between fat-1 mice and wildtype littermates raised and kept under identical conditions with regard to handling and diet. Furthermore, a study by Ji et al. reported the inability of fat-1 transgenic mice to generate homozygous offspring illustrating possible problems with using homozygous fat-1 mice [27]. Previous studies determined the fatty acid composition of various tissues from wildtype and fat-1 mice by showing relative n-6/n-3 PUFA ratios, combining different omega-3 and omega-6 fatty acids for the comparison, or by describing the fatty acid levels as relative percentages [2,3,6,28]. In this study, we extended this approach by quantifying total AA, EPA and DHA levels. AA and DHA were the major PUFAs in the assayed organs of wildtype and fat-1 mice. AA levels were significantly lower in all organs of fat-1 mice except the liver and lung, where differences missed significance. This is in line with our previous studies [22,29], and might be due to tissue specific fatty acid processing, given that the liver is the most important organ for fatty acid turnover and metabolism, while in the lung the rather sustained levels of arachidonic acid could be due to arachidonic acid-derived prostaglandins and leukotrienes being particularly involved in immune function in the lung. EPA was detected at low levels in all organs. A striking finding of the data presented here is the very constant ratio of DHA formation in the organs and tissues of fat-1 mice with fold differences between 1.4- to 1.7-fold compared to wildtype mice, which has, to our knowledge, not been described before. This is in contrast to the fold differences in EPA levels, which were highly heterogeneous ranging between 2.5- to 12.6-fold, which might be due to differences in the tissue-specific expression of elongase enzymes [30].

Lipid metabolites and mediators have been a scientific focus in the recent years with the discovery of their anti-inflammatory and pro-resolving properties [31,32,33]. Enzymatic actions through lipoxygenases as well as monooxygenase actions of several CYPs or non-enzymatic autooxidation lead to the formation of monohydroxy lipid metabolites, which amongst others act as precursors of resolvins, protectins, maresins and lipoxins. Assuming unselective metabolization of the fatty acids by these systems should lead to fold changes in lipid metabolites that are comparable to the fold changes observed for their parent fatty acids in the fat-1 mice. While the fold increase of DHA-derived HDHAs was broadly similar to the increase in DHA from wildtype to the fat-1 organs and tissues, the fold increase of HEPEs in fat-1 mice overtook the already marked increase of the parent fatty acid EPA. This underscores a high utilization of EPA for metabolite formation, which is in accordance with previous findings in rats as well as human volunteers supplemented with n-3 PUFA [24,25].

A recent study focused on the analysis of only the plasma lipidome of wildtype and fat-1 mice on an n-6 PUFA rich diet [34]. The most abundant changes in the plasma of fat-1 mice were observed in lipid metabolites derived from the CYP450 pathway. A small panel of monohydroxy lipid metabolites were also analyzed, of which 5-HEPE and 17-HDHA were found in significantly higher amounts, while 5-HETE and 8-HDHA levels were significantly lower in fat-1 mice. The predominant lipid metabolite in plasma was 12-HETE, which is consistent with our previous studies [16]. However, several important metabolites such as 18-HEPE and 14-HDHA were not described.

We expanded this approach here by analysis of monohydroxy lipid metabolites in a range of tissues and organs of wildtype and fat-1 mice, which has, to our knowledge, not been reported before. Here, we found specific patterns of metabolites in different organs: AA-derived HETEs followed by DHA-derived HDHAs made up the biggest relative content in most of the assayed organs, except for the wildtype muscle tissue and fat-1 kidney, muscle and heart tissue, where levels of assayed HDHAs were higher. In this context, the lung and spleen of fat-1 mice are particularly noteworthy with high HEPE formation, mainly 12-HEPE, overtaking the 14-HDHA dominated HDHA levels. 18-HEPE and 9-HEPE were the most prominent EPA metabolites in the other assayed fat-1 organs. A recent study demonstrated an anti-remodelling effect of 18-HEPE in heart failure [35]. While Endo et al. found approximately 30 pg 18-HEPE/heart in fat-1 mice, we found much higher levels of 18-HEPE in the heart (433 ng/g), a level only surpassed in the spleen in our study. This could be explained by the hydrolysis step employed in our study, which essentially liberates all fatty acids from ester bonds and thus detects free and esterified metabolites. While the elucidation of the enzymatic pathway for 18-HEPE formation remains the subject of current research, 18-hydroxylation of EPA is an important step in the formation of anti-inflammatory and pro-resolving E-resolvins.

High 12-hydroxy formation was observed in the lung and spleen with high 12-HEPE levels and even higher 12-HETE levels, possibly due to higher blood content and hence high expression of platelet 12-lipoxygenase in those organs. Interestingly, levels of the corresponding 14-HDHA were low in comparison, even though the parent fatty acid DHA was more prevalent than EPA. This points towards a higher affinity of platelet 12-LOX for EPA and AA in the lung and spleen. Future studies on the reaction kinetics of platelet 12-LOX for the formation of lipid metabolites from polyunsaturated fatty acids are needed. 14-HDHA as an important precursor in the formation of maresins was present at low levels in all assayed organs and tissues of wildtype and fat-1 mice.

15-lipoxygenation is an important step in the formation of anti-inflammatory lipid mediators. Of particular interest is the 15-LOX metabolite 17-HDHA, which acts as a precursor of D-resolvins and has also direct anti-inflammatory and pro-resolving effects [36,37]. 17-HDHA was detected in all assayed organs and tissues of wildtype and fat-1 mice with comparable levels in the colon and liver tissue to the ones observed in our previous studies [22,38]. 17-HDHA levels in the lung were very low, while 17-HDHA was one of the main DHA-derived metabolites in spleen, kidney, heart and muscle tissue. However, resolvin D_1_ and 10,17-DiHDHA were not detectable in the assayed samples with the protocol used here and its limits of detection.

One limitation of the data presented here is the focus on the analysis of total fatty acids (extracted directly from tissue homogenates) and total metabolites (prepared using hydrolysis of tissue homogenates). While this allows for a straightforward assessment as performed here it leaves several questions for future studies, particularly regarding the presence of fatty acids and metabolites in different lipid and protein classes—and we are currently working to establish these measurements.

## 5. Conclusions

Taken together, the data presented here provide a comprehensive overview on the tissue distribution of monohydroxy lipid metabolites derived from AA, EPA and DHA. Furthermore, this study showed a remarkably constant increase of DHA organ levels in fat-1 mice as compared to wildtype mice and strong utilization of the low EPA levels for HEPE formation. Additional studies are necessary to address the underlying mechanism for lipid metabolite formation, i.e., de-novo synthesis or release of preformed compounds from phospholipids as well as enzymatic or non-enzymatic formation, and to characterize enzymes involved in their formation, release, downstream processing and their biological effects. Furthermore, future work will have to address a direct comparison of lipid metabolites in fat-1 mice with the effect of dietary n-3 PUFA supplementation.

## Figures and Tables

**Table 1:** Polyunsaturated fatty acid levels in wildtype and fat-1 mice.

Organ	Fatty Acid	Wildtype (μg/g)	Fat-1 (μg/g)	*p*-Value	AA Difference (-Fold)	EPA Difference (-Fold)	DHA Difference (-Fold)
Colon	AA	1470 ± 41.0	1023 ± 108	0.001	0.70		
	EPA	36.0 ± 4.7	171.2 ± 21.7	<0.001		4.8	
	DHA	239.7 ± 11.2	380.7 ± 42.1	0.003			1.6
Liver	AA	4448 ± 94.9	4152 ± 225	0.2	0.93		
	EPA	47.3 ± 17.9	116.5 ± 8.1	0.02		2.5	
	DHA	1147 ± 62.9	1883 ± 80.1	<0.001			1.6
Lung	AA	2154 ± 71.2	1714 ± 199	0.06	0.80		
	EPA	20.8 ± 3.0	96.7 ± 17.6	0.001		4.6	
	DHA	415.7 ± 17.9	692.7 ± 59.2	0.001			1.7
Spleen	AA	2195 ± 173.7	1444 ± 234.5	0.03	0.66		
	EPA	13.4 ± 0.8	169.2 ± 29.0	<0.001		12.6	
	DHA	359.8 ± 22.7	538.9 ± 88.6	0.03			1.5
Heart	AA	2574 ± 93	1942 ± 105	0.002	0.75		
	EPA	15.3 ± 1.9	51.37 ± 5.8	<0.001		3.4	
	DHA	2689 ± 109	4340 ± 126	<0.001			1.6
Muscle	AA	1039 ± 14.3	442.5 ± 32.5	<0.001	0.43		
	EPA	9.9 ± 1.4	54.7 ± 3.1	<0.001		5.5	
	DHA	913.9 ± 25.2	1420 ± 66.7	<0.001			1.6
Kidney	AA	3556 ± 134	2947 ± 132	0.02	0.83		
	EPA	13.8 ± 1.4	172.3 ± 16.3	<0.001		12.4	
	DHA	2304 ± 54.8	3110 ± 114	<0.001			1.4

AA, EPA and DHA levels in the colon, liver, lung, spleen, heart, skeletal muscle and kidney of wildtype (*n* = 7) and fat-1 transgenic mice (*n* = 4). Shown are the means ± SEM (μg/g) and fold differences. *p*-values versus wildtype mice are as indicated.

**Table 2:** Total monohydroxy lipid metabolite levels in wildtype and fat-1 mice.

Organ	Lipid Metabolites	Wildtype (ng/g)	Fat-1 (ng/g)	*p*-Value	HETE Difference (-Fold)	HEPE difference (-Fold)	HDHA Difference (-Fold)
Colon	HETE	4260 ± 282	2707 ± 249	0.005	0.64		
	HEPE	61.0 ± 7.8	1340 ± 94.2	<0.001		21.9	
	HDHA	746.1 ± 42.6	1183 ± 103	0.001			1.6
Liver	HETE	6336 ± 698	4940 ± 298	0.18	0.78		
	HEPE	36.0 ± 4.4	489.0 ± 90.4	<0.001		13.6	
	HDHA	1586 ± 186	2543 ± 168	0.008			1.6
Lung	HETE	16,362 ± 1641	17,290 ± 1926	0.73	1.06		
	HEPE	156.1 ± 24.5	3057 ± 530	<0.001		19.6	
	HDHA	740.6 ± 102	1629 ± 152	<0.001			2.2
Spleen	HETE	86,511 ± 3888	58,515 ± 2095	<0.001	0.68		
	HEPE	403.2 ± 63.2	17,711 ± 2181	<0.001		43.9	
	HDHA	5995 ± 791	7425 ± 485	0.24			1.2
Heart	HETE	8094 ± 845	9826 ± 3023	0.50	1.21		
	HEPE	36.6 ± 10	885.9 ± 163	<0.001		24.2	
	HDHA	6642 ± 960	22,855 ± 8748	0.03			3.4
Muscle	HETE	2471 ± 234	1057 ± 129	0.002	0.43		
	HEPE	19.4 ± 2.4	156.9 ± 20.9	<0.001		8.1	
	HDHA	3759 ± 347	4021 ± 459	0.66			1.1
Kidney	HETE	5487 ± 719	5339 ± 1541	0.92	0.97		
	HEPE	40.3 ± 6.6	919.2 ± 417	0.02		22.8	
	HDHA	5108 ± 832	7896 ± 2509	0.22			1.6

Total monohydroxy lipid metabolite levels in the colon, liver, lung, spleen, heart, skeletal muscle and kidney of wildtype and fat-1 mice. Total HETE level is given by the sum of 5-HETE, 8-HETE, 9-HETE, 11-HETE, 12-HETE and 15-HETE. Total HEPE level is made up of 5-HEPE, 8-HEPE, 9-HEPE, 12-HEPE, 15-HEPE and 18-HEPE. Total HDHA level is the sum of 4-HDHA, 7-HDHA, 8-HDHA, 10-HDHA, 11-HDHA, 13-HDHA, 14-HDHA, 16-HDHA, 17-HDHA, 20-HDHA. Shown are the means ± SEM (ng/g) and fold differences. *p*-values versus wildtype mice are as indicated.

## Supplementary Materials

The following are available online at www.mdpi.com/2079-7737/6/1/9/s1, Figure S1: Standard curves for the assayed fatty acids, (A) arachidonic acid (AA); (B) eicosapentaenoic acid (EPA); and (C) docosahexaenoic acid (DHA). Calibrators used were 1, 5, 10, 20, 50, 100 and 200 μg of fatty acid, Figure S2: Standard curves for the assayed lipid metabolites (A) hydroxyeicosatetraenoic acids (HETE); (B) hydroxyeicosapentaenoic acids (HEPE); (C) hydroxydocosahexaenoic (HDHA) acids; and (D) resolvin D1 and 10,17-DiHDHA. Calibrators used were 1, 2, 5, 10 and 20 ng of respective compounds, Table S1: Multiple reaction monitoring characterization of the measured compounds, Table S2: Monohydroxy lipid metabolite levels (ng/g) in colon of wildtype and fat-1 mice, Table S3: Monohydroxy lipid metabolite levels (ng/g) in liver of wildtype and fat-1 mice, Table S4: Monohydroxy lipid metabolite levels (ng/g) in lung of wildtype and fat-1 mice, Table S5: Monohydroxy lipid metabolite levels (ng/g) in spleen of wildtype and fat-1 mice, Table S6: Monohydroxy lipid metabolite levels (ng/g) in heart of wildtype and fat-1 mice, Table S7: Monohydroxy lipid metabolite levels (ng/g) in skeletal muscle of wildtype and fat-1 mice, Table S8: Monohydroxy lipid metabolite levels (ng/g) in kidney of wildtype and fat-1 mice.
