# Supplementary material for: Quantitative Profiling of Hydroxy Lipid Metabolites in Mouse Organs Reveals Distinct Lipidomic Profiles and Modifications Due to Elevated n-3 Fatty Acid Levels
Source: Biology (Basel). 2017 Feb 4;6(1):9. doi: 10.3390/biology6010009 (PMC5372002; doi:10.3390/biology6010009)
Supplement: Supplementary file 1 [file biology-06-00009-s001.pdf]

# Supplementary Materials: Quantitative Profiling of Hydroxy Lipid Metabolites in Mouse Organs Reveals Distinct Lipidomic Profiles and Modifications Due to Elevated n-3 Fatty Acid Levels

**Cheng-Ying Chiu, Christopher Smyl, Inci Dogan, Michael Rothe and Karsten-H Weylandt**

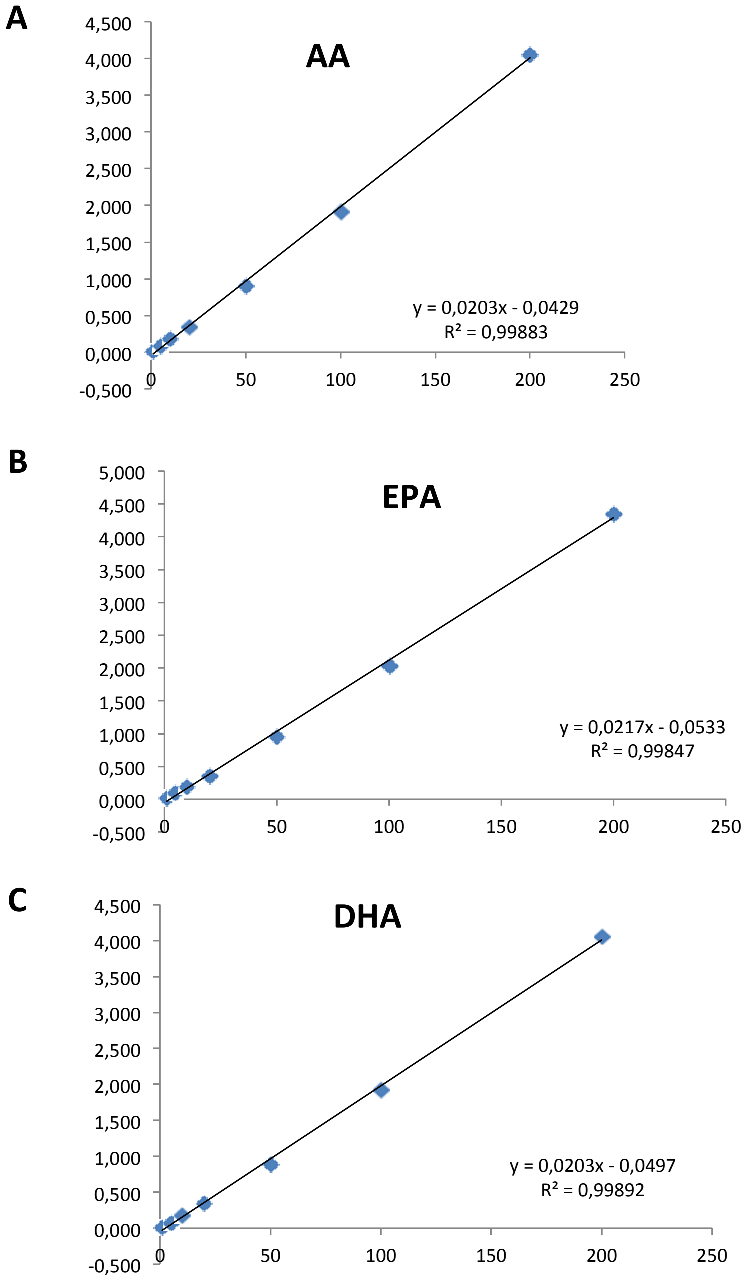


**Figure S1.** Standard curves for the assayed fatty acids, (**A**) arachidonic acid (AA); (**B**) eicosapentaenoic acid (EPA); and (**C**) docosahexaenoic acid (DHA). Calibrators used were 1, 5, 10, 20, 50, 100 and 200 μg of fatty acid.

5-HETE - 5 Levels, 5 Levels Used, 5 Points, 5 Points Used, 0 QCs

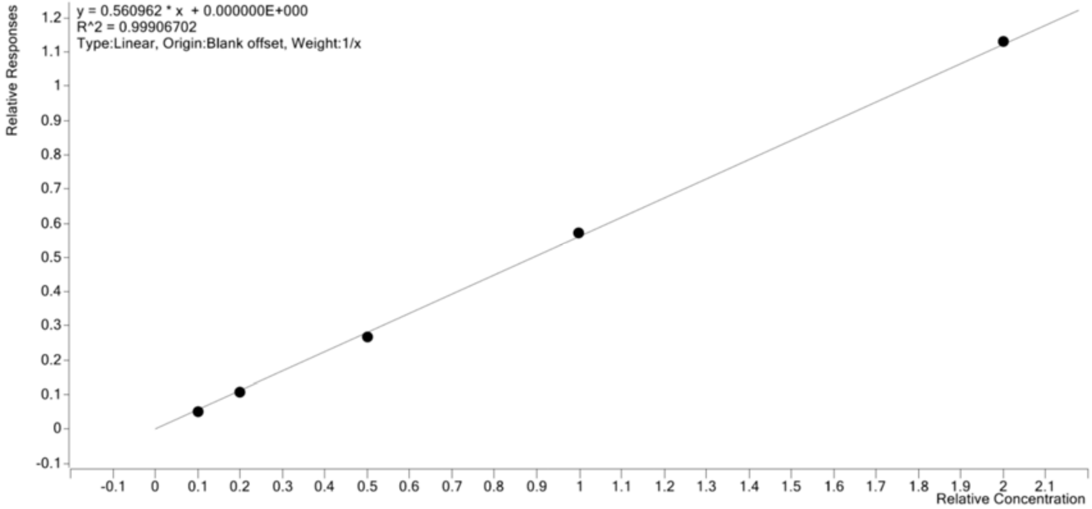


8-HETE - 5 Levels, 5 Levels Used, 5 Points, 5 Points Used, 0 QCs

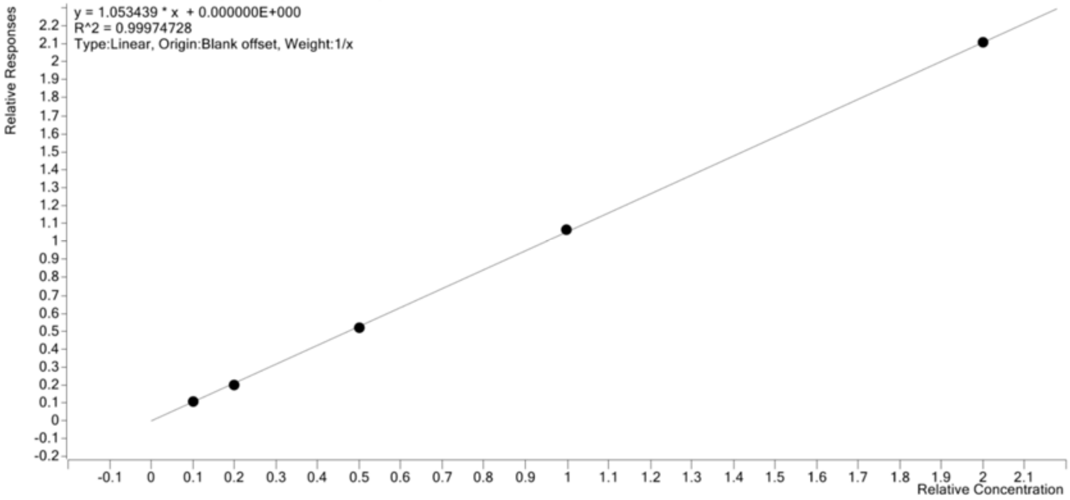


9-HETE - 5 Levels, 5 Levels Used, 5 Points, 5 Points Used, 0 QCs

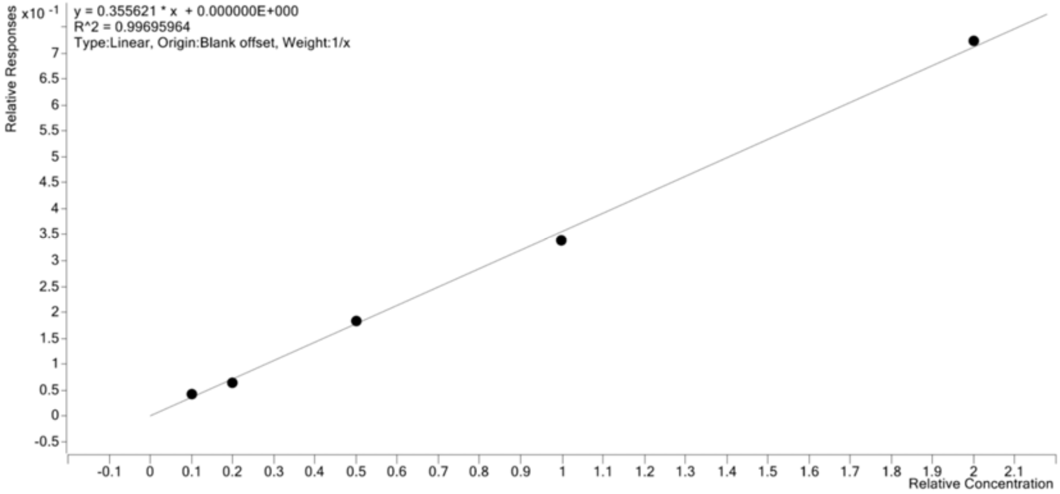

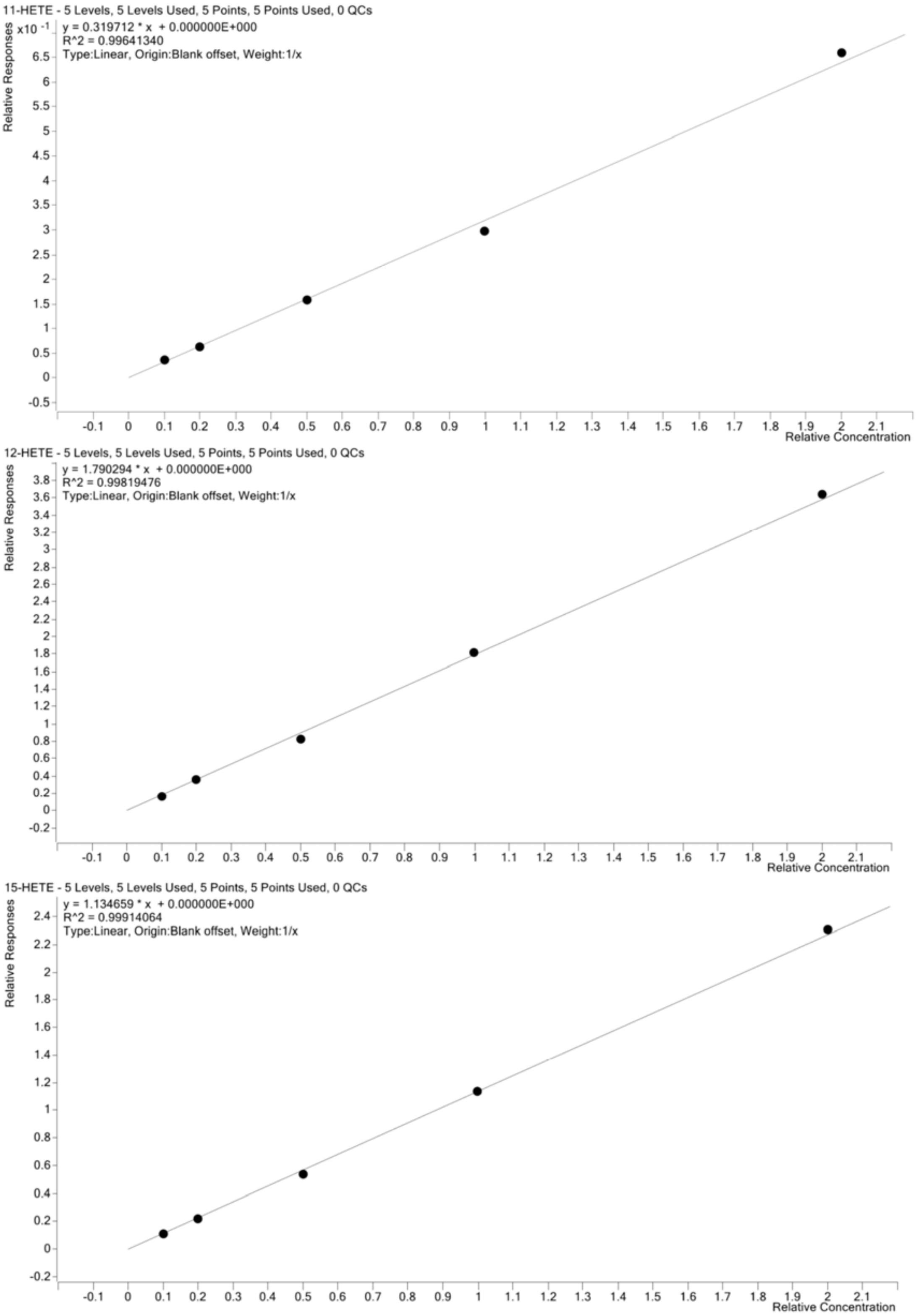
11-HETE - 5 Levels, 5 Levels Used, 5 Points, 5 Points Used, 0 QCs
x10 -1
y = 0.319712 * x + 0.000000E+000
R^2 = 0.99641340
Type:Linear, Origin:Blank offset, Weight:1/x
Relative Responses
Relative Concentration
12-HETE - 5 Levels, 5 Levels Used, 5 Points, 5 Points Used, 0 QCs
y = 1.790294 * x + 0.000000E+000
R^2 = 0.99819476
Type:Linear, Origin:Blank offset, Weight:1/x
Relative Responses
Relative Concentration
15-HETE - 5 Levels, 5 Levels Used, 5 Points, 5 Points Used, 0 QCs
y = 1.134659 * x + 0.000000E+000
R^2 = 0.99914064
Type:Linear, Origin:Blank offset, Weight:1/x
Relative Responses
Relative Concentration

(**A**) HETE

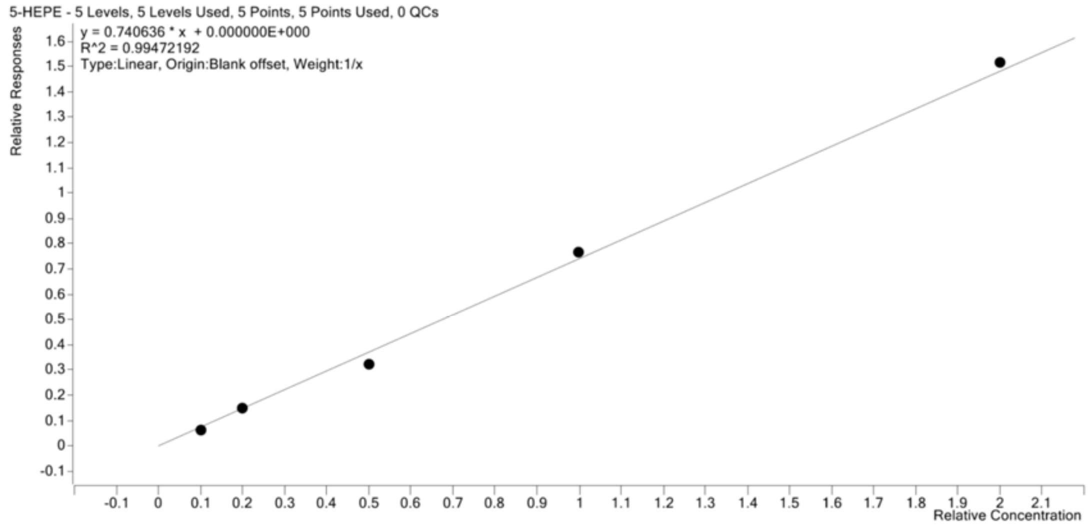
5-HEPE - 5 Levels, 5 Levels Used, 5 Points, 5 Points Used, 0 QCs
y = 0.740636 * x + 0.000000E+000
R^2 = 0.99472192
Type:Linear, Origin:Blank offset, Weight:1/x
Relative Responses
Relative Concentration

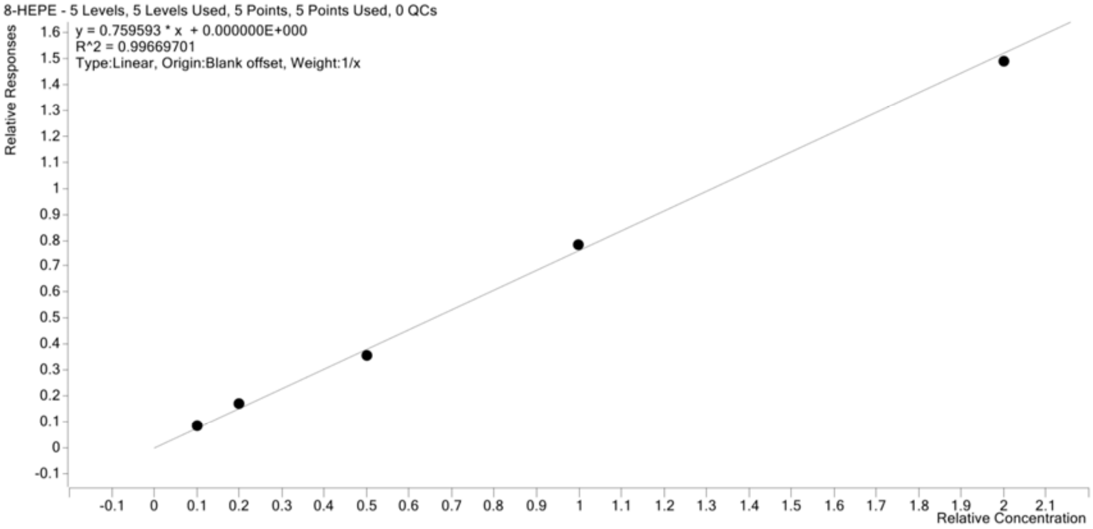
8-HEPE - 5 Levels, 5 Levels Used, 5 Points, 5 Points Used, 0 QCs
y = 0.759593 * x + 0.000000E+000
R^2 = 0.99669701
Type:Linear, Origin:Blank offset, Weight:1/x
Relative Responses
Relative Concentration

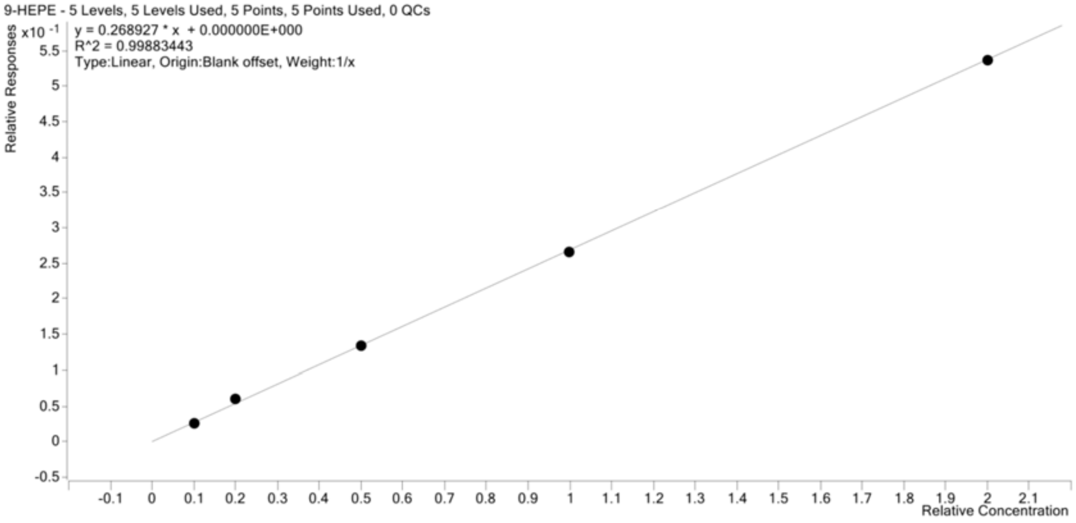
9-HEPE - 5 Levels, 5 Levels Used, 5 Points, 5 Points Used, 0 QCs
x10 -1
y = 0.268927 * x + 0.000000E+000
R^2 = 0.99883443
Type:Linear, Origin:Blank offset, Weight:1/x
Relative Responses
Relative Concentration

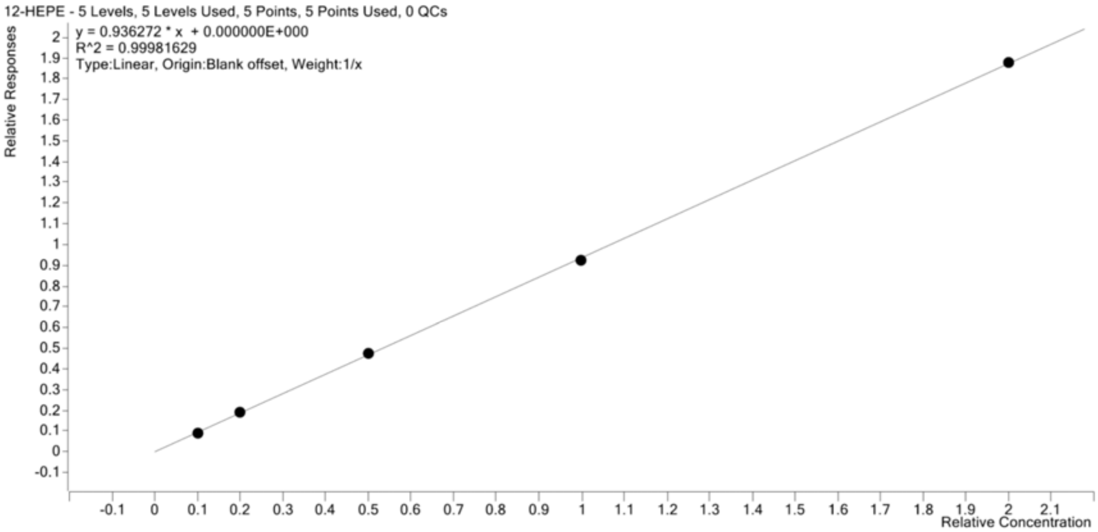
12-HEPE - 5 Levels, 5 Levels Used, 5 Points, 5 Points Used, 0 QCs
y = 0.936272 * x + 0.000000E+000
R^2 = 0.99981629
Type:Linear, Origin:Blank offset, Weight:1/x
Relative Responses
Relative Concentration

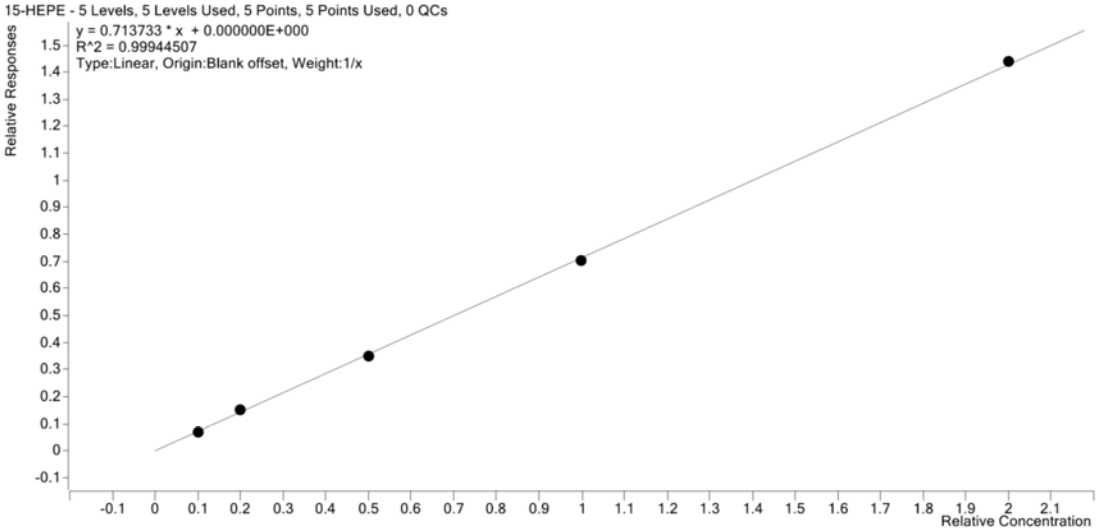
15-HEPE - 5 Levels, 5 Levels Used, 5 Points, 5 Points Used, 0 QCs
y = 0.713733 * x + 0.000000E+000
R^2 = 0.99944507
Type:Linear, Origin:Blank offset, Weight:1/x
Relative Responses
Relative Concentration

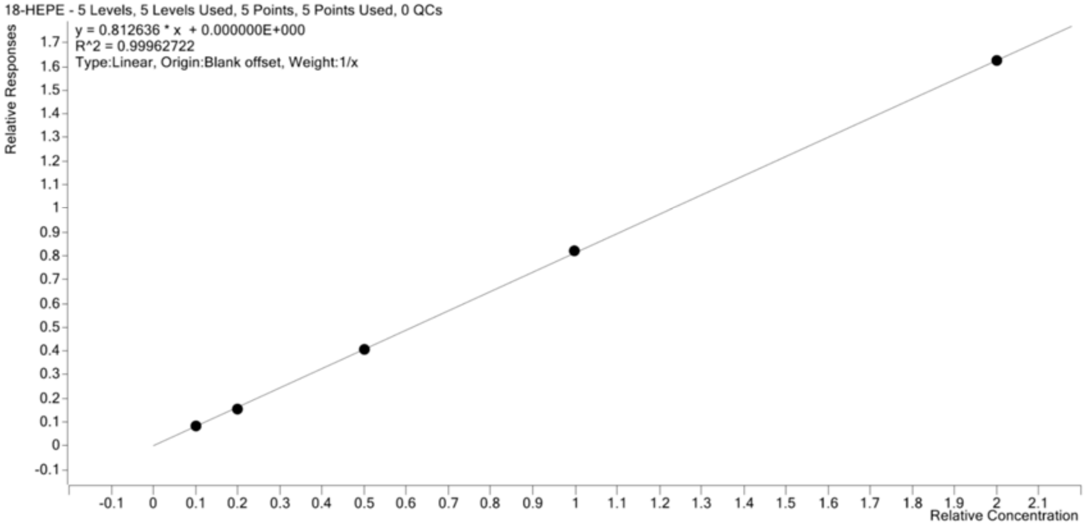
18-HEPE - 5 Levels, 5 Levels Used, 5 Points, 5 Points Used, 0 QCs
y = 0.812636 * x + 0.000000E+000
R^2 = 0.99962722
Type:Linear, Origin:Blank offset, Weight:1/x
Relative Responses
Relative Concentration

(**B**) **HEPE**

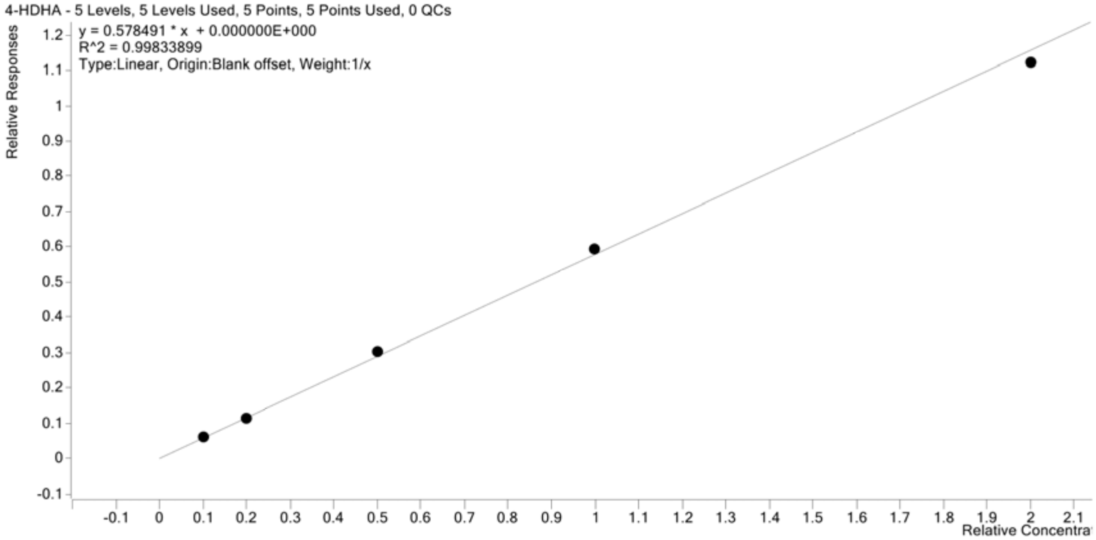
4-HDHA - 5 Levels, 5 Levels Used, 5 Points, 5 Points Used, 0 QCs
y = 0.578491 * x + 0.000000E+000
R^2 = 0.99833899
Type:Linear, Origin:Blank offset, Weight:1/x
Relative Responses
Relative Concentra

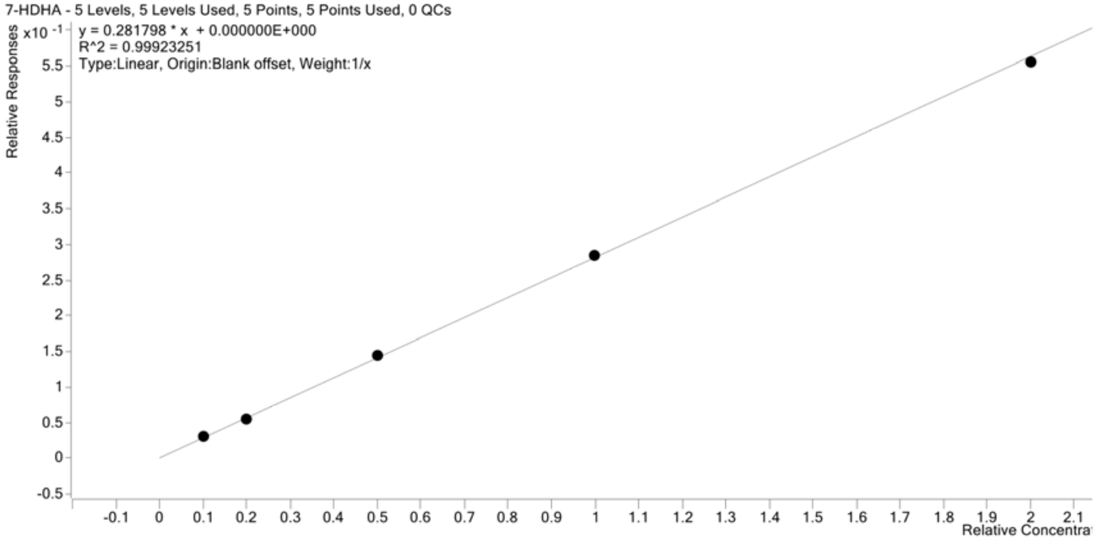
7-HDHA - 5 Levels, 5 Levels Used, 5 Points, 5 Points Used, 0 QCs
x10 -1
y = 0.281798 * x + 0.000000E+000
R^2 = 0.99923251
Type:Linear, Origin:Blank offset, Weight:1/x
Relative Responses
Relative Concentra

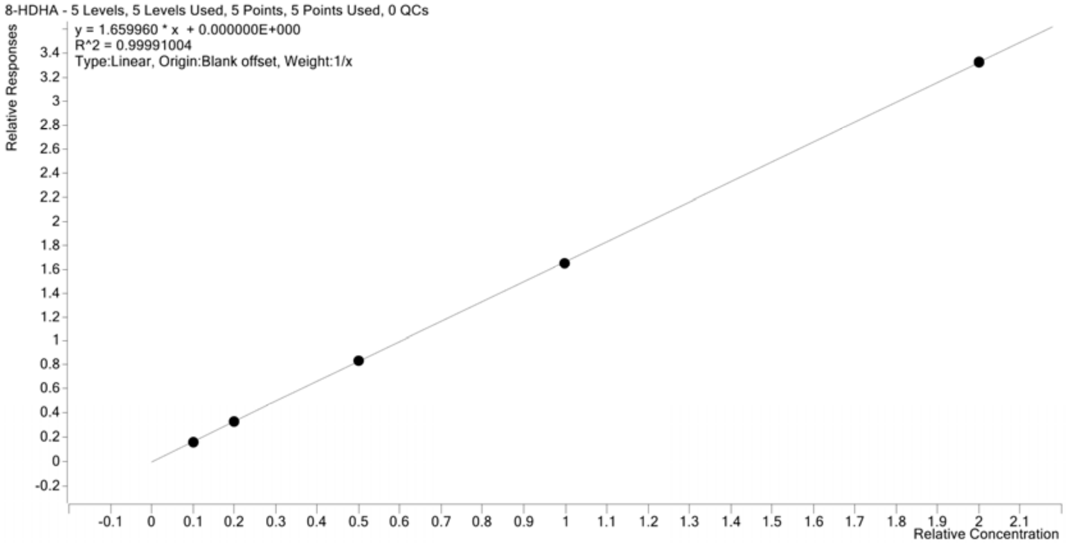
8-HDHA - 5 Levels, 5 Levels Used, 5 Points, 5 Points Used, 0 QCs
y = 1.659960 * x + 0.000000E+000
R^2 = 0.99991004
Type:Linear, Origin:Blank offset, Weight:1/x
Relative Responses
Relative Concentration

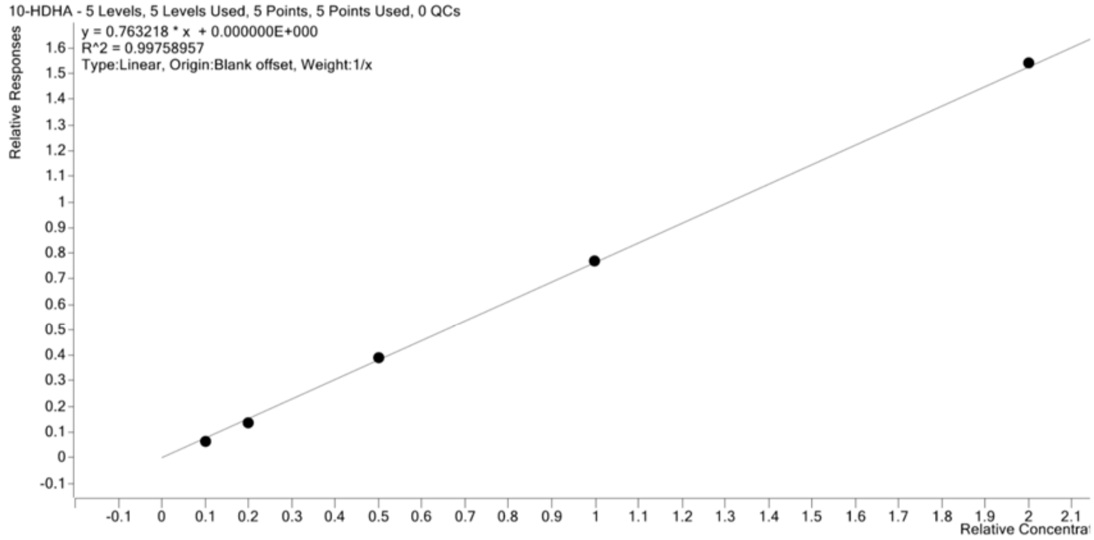
10-HDHA - 5 Levels, 5 Levels Used, 5 Points, 5 Points Used, 0 QCs
y = 0.763218 * x + 0.000000E+000
R^2 = 0.99758957
Type:Linear, Origin:Blank offset, Weight:1/x
Relative Responses
Relative Concentrat

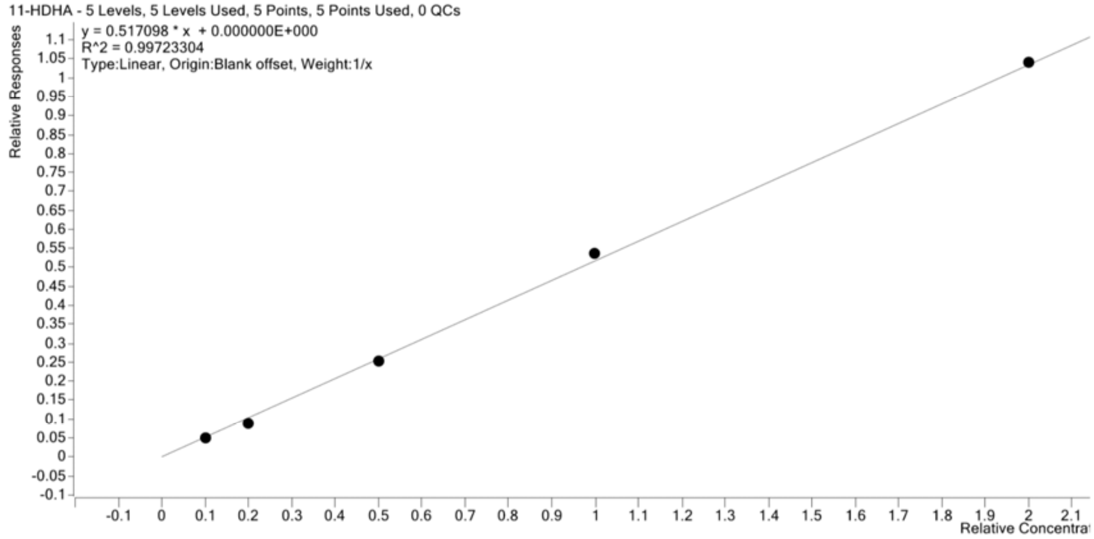
11-HDHA - 5 Levels, 5 Levels Used, 5 Points, 5 Points Used, 0 QCs
y = 0.517098 * x + 0.000000E+000
R^2 = 0.99723304
Type:Linear, Origin:Blank offset, Weight:1/x
Relative Responses
Relative Concentrat

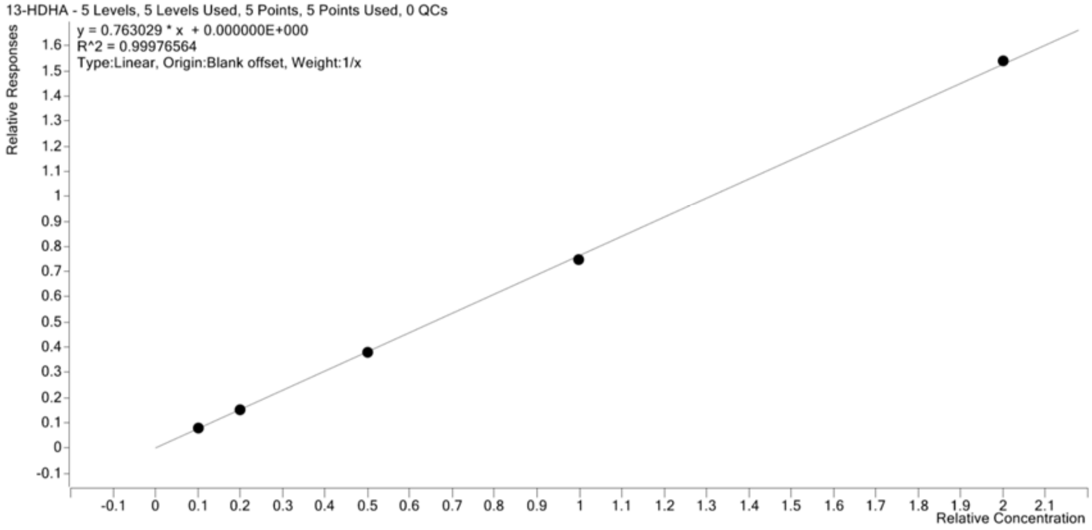
13-HDHA - 5 Levels, 5 Levels Used, 5 Points, 5 Points Used, 0 QCs
y = 0.763029 * x + 0.000000E+000
R^2 = 0.99976564
Type:Linear, Origin:Blank offset, Weight:1/x
Relative Responses
Relative Concentration

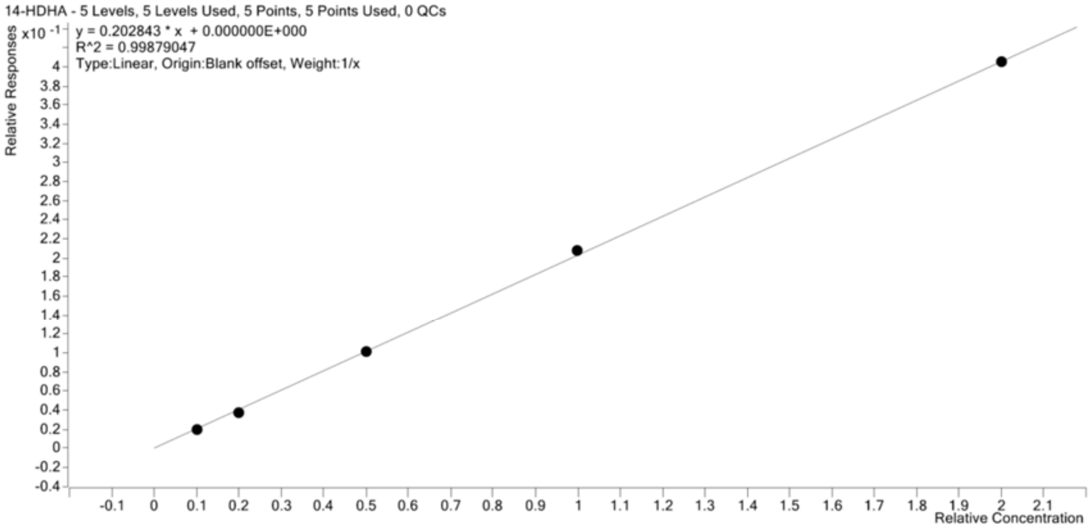
14-HDHA - 5 Levels, 5 Levels Used, 5 Points, 5 Points Used, 0 QCs
y = 0.202843 * x + 0.000000E+000
R^2 = 0.99879047
Type:Linear, Origin:Blank offset, Weight:1/x
x10 -1
Relative Responses
Relative Concentration

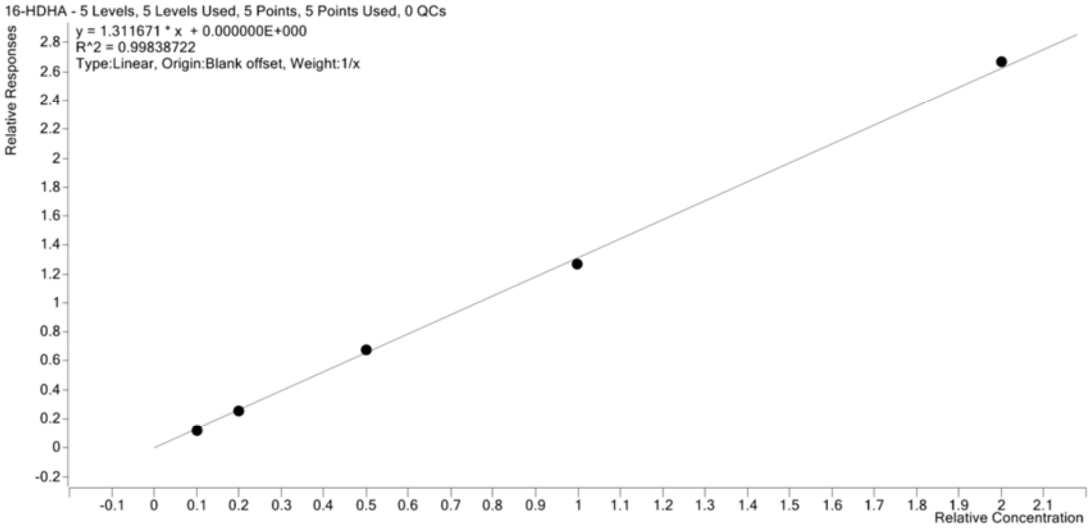
16-HDHA - 5 Levels, 5 Levels Used, 5 Points, 5 Points Used, 0 QCs
y = 1.311671 * x + 0.000000E+000
R^2 = 0.99838722
Type:Linear, Origin:Blank offset, Weight:1/x
Relative Responses
Relative Concentration

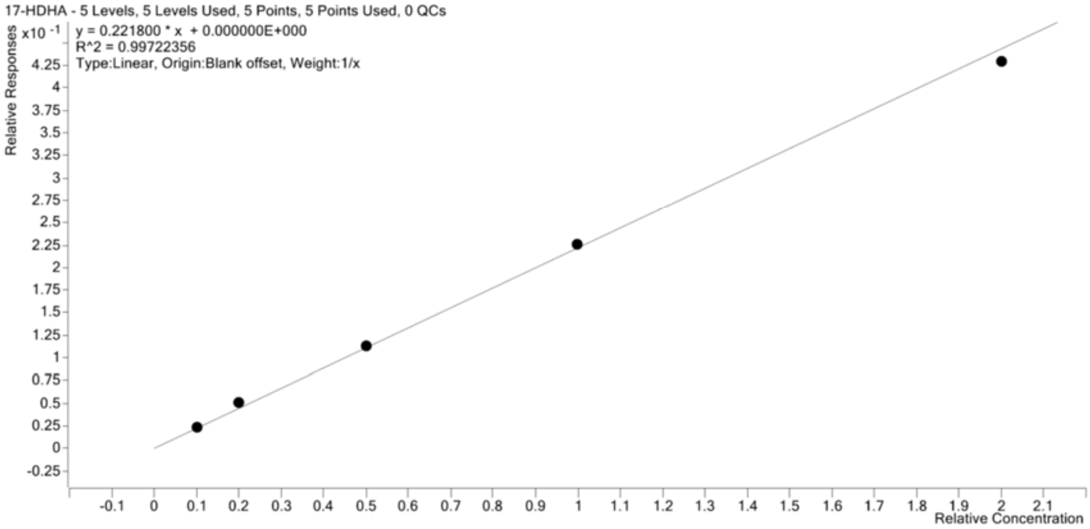
17-HDHA - 5 Levels, 5 Levels Used, 5 Points, 5 Points Used, 0 QCs
y = 0.221800 * x + 0.000000E+000
R^2 = 0.99722356
Type:Linear, Origin:Blank offset, Weight:1/x
x10 -1
Relative Responses
Relative Concentration

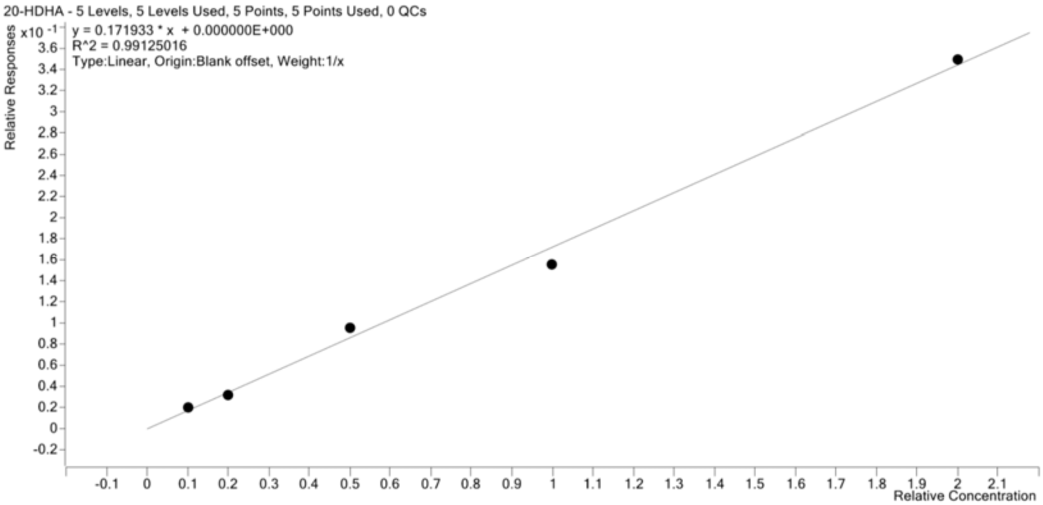


(**C**) HDHA

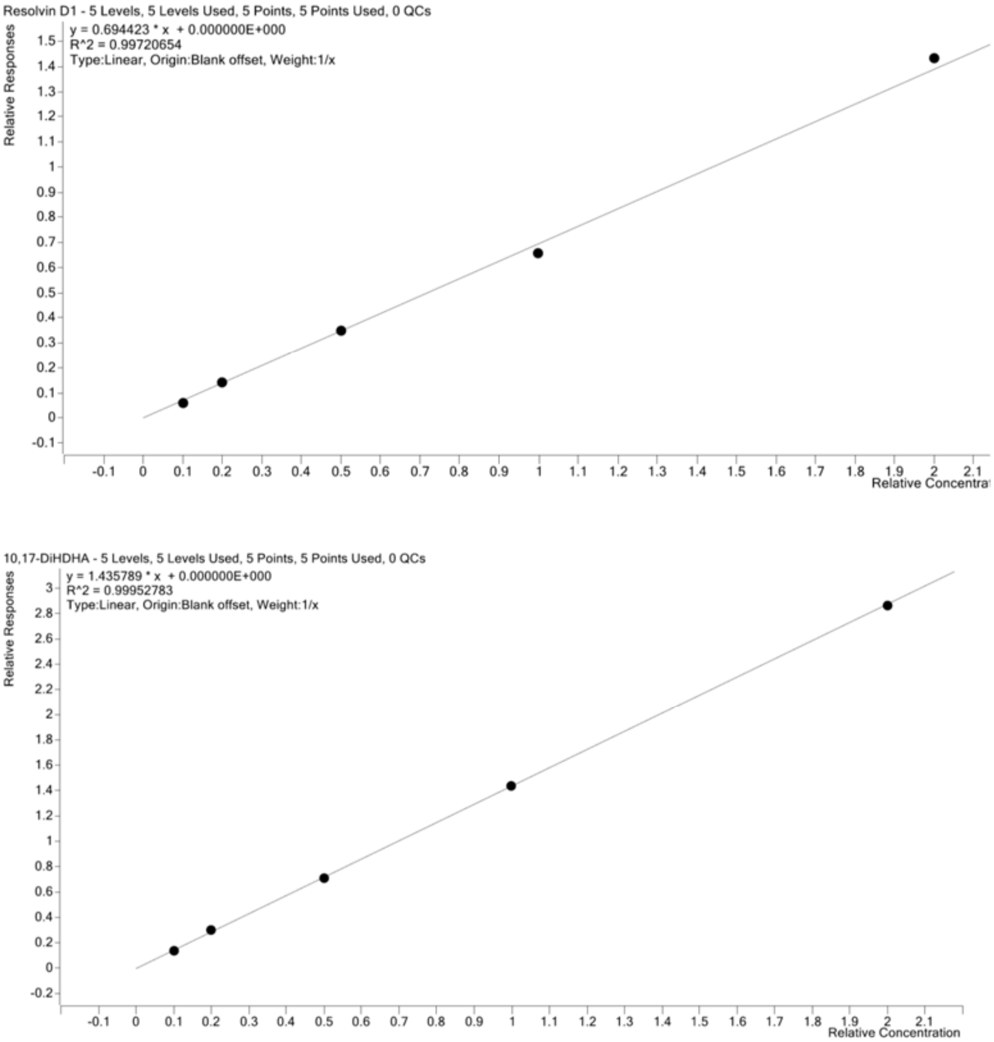


(**D**) Resolvin D1, 10,17-DiHDHA

**Figure S2.** Standard curves for the assayed lipid metabolites (**A**) hydroxyeicosatetraenoic acids (HETE); (**B**) hydroxyeicosapentaenoic acids (HEPE); (**C**) hydroxydocosahexaenoic (HDHA) acids; and (**D**) resolvin D1 and 10,17-DiHDHA. Calibrators used were 1, 2, 5, 10 and 20 ng of respective compounds.

**Table S1.** Multiple reaction monitoring characterization of the measured compounds.

| Compound | Precursor Ion | Product Ion | Fragmentor Voltage | Collision Energy [V] | Cell Accel. Voltage [V] | Ret Time (min) |
|---|---|---|---|---|---|---|
| 12-HEPE | 317.2 | 179.1 | 120 | 5 | 1 | 9.93 |
| 15-HEPE | 317.2 | 219.1 | 120 | 5 | 1 | 9.58 |
| 18-HEPE | 317.2 | 259.1 | 140 | 3 | 5 | 9.12 |
| 5-HEPE | 317.2 | 115.1 | 110 | 6 | 1 | 10.12 |
| 8-HEPE | 317.2 | 155.1 | 110 | 7 | 2 | 9.77 |
| 9-HEPE | 317.2 | 167.1 | 110 | 5 | 1 | 9.99 |
| 11-HETE | 319.2 | 149.1 | 130 | 17 | 1 | 11.30 |
| 12-HETE | 319.2 | 179.1 | 110 | 5 | 1 | 11.62 |
| 15-HETE | 319.2 | 219.1 | 140 | 4 | 8 | 10.88 |
| 5-HETE | 319.2 | 115.1 | 110 | 10 | 1 | 12.00 |
| 8-HETE | 319.2 | 155.1 | 120 | 8 | 1 | 11.53 |
| 9-HETE | 319.2 | 151.1 | 130 | 5 | 2 | 11.83 |
| 15-HETE-d8 | 327.2 | 226.1 | 140 | 4 | 1 | 10.73 |
| 10-HDHA | 343.2 | 153.1 | 110 | 9 | 1 | 11.43 |
| 11-HDHA | 343.2 | 149.1 | 110 | 5 | 1 | 11.63 |
| 13-HDHA | 343.2 | 193.1 | 110 | 7 | 1 | 11.23 |
| 14-HDHA | 343.2 | 205.1 | 110 | 6 | 2 | 11.43 |
| 16-HDHA | 343.2 | 233.1 | 130 | 6 | 5 | 11.00 |
| 17-HDHA | 343.2 | 201.1 | 110 | 6 | 6 | 11.07 |
| 20-HDHA | 343.2 | 241.1 | 130 | 5 | 3 | 10.55 |
| 4-HDHA | 343.2 | 101.1 | 130 | 7 | 2 | 12.53 |
| 7-HDHA | 343.2 | 141.1 | 110 | 4 | 1 | 11.75 |
| 8-HDHA | 343.2 | 189.1 | 110 | 4 | 2 | 11.90 |
| 10,17-DIHDHA | 359.2 | 153.1 | 90 | 10 | 8 | 7.42 |
| Rv D1 | 375.2 | 141.1 | 110 | 9 | 1 | 6.27 |

**Table S2.** Monohydroxy lipid metabolite levels (ng/g) in colon of wildtype and fat-1 mice.

| | Colon | | | |
|---|---|---|---|---|
| | **Wild Type** | **Fat-1** | ***p*-Value** | **Change (-Fold)** |
| 5-HETE | 447.43 ± 48.10 | 307.63 ± 36.66 | 0.078 | 0.69 |
| 8-HETE | 128.43 ± 46.85 | 110.23 ± 56.76 | 0.815 | 0.86 |
| 9-HETE | 120.30 ± 16.16 | 70.83 ± 5.67 | 0.053 | 0.59 |
| 11-HETE | 359.21 ± 37.07 | 247.75 ± 51.45 | 0.108 | 0.69 |
| 12-HETE | 800.59 ± 79.17 | 406.18 ± 25.73 | 0.006 | 0.51 |
| 15-HETE | 2403.80 ± 180.52 | 1564.48 ± 170.00 | 0.013 | 0.65 |
| 5-HEPE | 4.26 ± 1.15 | 91.73 ± 12.33 | <0.001 | 21.55 |
| 8-HEPE | 5.31 ± 1.42 | 56.675 ± 8.79 | <0.001 | 10.66 |
| 9-HEPE | 16.93 ± 7.10 | 580.03 ± 105.26 | <0.001 | 34.26 |
| 12-HEPE | 12.17 ± 2.13 | 107.28 ± 16.81 | <0.001 | 8.81 |
| 15-HEPE | 6.67 ± 1.20 | 99.25 ± 13.43 | <0.001 | 14.88 |
| 18-HEPE | 15.63 ± 3.12 | 405.03 ± 21.28 | <0.001 | 25.92 |
| 4-HDHA | 62.00 ± 5.72 | 95.10 ± 12.52 | 0.022 | 1.53 |
| 7-HDHA | 21.79 ± 1.64 | 31.38 ± 2.77 | 0.011 | 1.44 |
| 8-HDHA | 6.70 ± 1.56 | 7.90 ± 0.35 | 0.586 | 1.18 |
| 10-HDHA | 17.61 ± 1.03 | 29.68 ± 3.52 | 0.002 | 1.68 |
| 11-HDHA | 9.64 ± 2.43 | 20.23 ± 4.00 | 0.039 | 2.10 |
| 13-HDHA | 62.30 ± 5.34 | 99.00 ± 11.56 | 0.009 | 1.59 |
| 14-HDHA | 63.79 ± 7.77 | 113.15 ± 9.29 | 0.003 | 1.77 |
| 16-HDHA | 122.93 ± 7.37 | 187.45 ± 23.00 | 0.009 | 1.52 |
| 17-HDHA | 139.33 ± 12.38 | 217.88 ± 28.74 | 0.016 | 1.56 |
| 20-HDHA | 240.03 ± 16.98 | 381.43 ± 30.75 | 0.002 | 1.59 |

**Table S3.** Monohydroxy lipid metabolite levels (ng/g) in liver of wildtype and fat-1 mice.

| | Liver | | | |
|---|---|---|---|---|
| | **Wild Type** | **Fat-1** | ***p*-Value** | **Change (-fold)** |
| 5-HETE | 1176.06 ± 173.90 | 841.53 ± 95.23 | 0.206 | 0.72 |
| 8-HETE | 625.86 ± 69.90 | 454.53 ± 54.20 | 0.129 | 0.73 |
| 9-HETE | 442.17 ± 58.28 | 328.10 ± 49.93 | 0.221 | 0.74 |
| 11-HETE | 236.37 ± 44.24 | 198.93 ± 39.01 | 0.586 | 0.84 |
| 12-HETE | 861.40 ± 116.64 | 589.40 ± 61.98 | 0.132 | 0.68 |
| 15-HETE | 2993.99 ± 259.15 | 2527.98 ± 209.38 | 0.254 | 0.84 |
| 5-HEPE | 8.89 ± 1.59 | 88.75 ± 3.21 | <0.001 | 9.99 |
| 8-HEPE | 3.14 ± 1.63 | 32.75 ± 2.35 | <0.001 | 10.42 |
| 9-HEPE | 0.00 | 124.23 ± 63.99 | 0.025 | |
| 12-HEPE | 4.63 ± 1.20 | 39.90 ± 7.13 | <0.001 | 8.62 |
| 15-HEPE | 5.24 ± 2.07 | 27.75 ± 5.12 | <0.001 | 5.29 |
| 18-HEPE | 14.13 ± 1.63 | 175.65 ± 24.10 | <0.001 | 12.43 |
| 4-HDHA | 232.84 ± 34.51 | 333.75 ± 12.00 | 0.063 | 1.43 |
| 7-HDHA | 91.50 ± 11.49 | 131.25 ± 6.36 | 0.037 | 1.43 |
| 8-HDHA | 33.81 ± 4.68 | 63.40 ± 6.09 | 0.004 | 1.87 |
| 10-HDHA | 71.71 ± 8.71 | 123.35 ± 14.42 | 0.010 | 1.72 |
| 11-HDHA | 70.37 ± 13.57 | 109.13 ± 12.13 | 0.090 | 1.55 |
| 13-HDHA | 110.50 ± 15.27 | 181.65 ± 14.97 | 0.014 | 1.64 |
| 14-HDHA | 179.87 ± 23.30 | 308.80 ± 25.87 | 0.007 | 1.72 |
| 16-HDHA | 147.61 ± 16.87 | 244.40 ± 22.12 | 0.007 | 1.66 |
| 17-HDHA | 230.36 ± 28.23 | 363.45 ± 34.77 | 0.017 | 1.58 |
| 20-HDHA | 417.00 ± 46.47 | 684.30 ± 42.93 | 0.004 | 1.64 |

**Table S4.** Monohydroxy lipid metabolite levels (ng/g) in lung of wildtype and fat-1 mice.

| | Lung | | | |
|---|---|---|---|---|
| | **Wild Type** | **Fat-1** | ***p*-Value** | **Change (-Fold)** |
| 5-HETE | 164.47 ± 11.17 | 144.63 ± 7.15 | 0.234 | 0.88 |
| 8-HETE | 257.50 ± 70.89 | 171.43 ± 15.23 | 0.390 | 0.67 |
| 9-HETE | 650.84 ± 65.00 | 691.08 ± 73.33 | 0.799 | 1.06 |
| 11-HETE | 1154.61 ± 115.02 | 1062.95 ± 97.96 | 0.508 | 0.92 |
| 12-HETE | 13,305.86 ± 1430.19 | 14,505.28 ± 1745.70 | 0.703 | 1.09 |
| 15-HETE | 828.54 ± 43.68 | 714.38 ± 84.59 | 0.268 | 0.86 |
| 5-HEPE | 0.57 ± 0.23 | 15.53 ± 3.71 | <0.001 | 27.17 |
| 8-HEPE | 0.63 ± 0.42 | 12.48 ± 1.93 | <0.001 | 19.85 |
| 9-HEPE | 10.41 ± 2.79 | 121.13 ± 16.73 | <0.001 | 11.63 |
| 12-HEPE | 142.71 ± 21.95 | 2869.40 ± 503.52 | <0.001 | 20.11 |
| 15-HEPE | 1.20 ± 0.58 | 22.90 ± 7.30 | 0.006 | 19.08 |
| 18-HEPE | 0.61 ± 0.61 | 15.23 ± 3.89 | 0.002 | 24.78 |
| 4-HDHA | 10.67 ± 2.54 | 22.93 ± 4.54 | 0.050 | 2.15 |
| 7-HDHA | 4.06 ± 0.79 | 6.15 ± 0.43 | 0.146 | 1.52 |
| 8-HDHA | 43.94 ± 7.15 | 105.90 ± 8.77 | 0.001 | 2.41 |
| 10-HDHA | 15.66 ± 1.50 | 28.90 ± 3.14 | 0.004 | 1.85 |
| 11-HDHA | 15.94 ± 2.93 | 41.35 ± 2.64 | <0.001 | 2.59 |
| 13-HDHA | 43.80 ± 8.45 | 84.45 ± 8.67 | 0.024 | 1.93 |
| 14-HDHA | 491.19 ± 73.19 | 1132.30 ± 120.83 | 0.002 | 2.31 |
| 16-HDHA | 22.39 ± 2.72 | 44.45 ± 3.59 | 0.002 | 1.99 |
| 17-HDHA | 62.36 ± 9.48 | 98.95 ± 13.40 | 0.041 | 1.59 |
| 20-HDHA | 30.59 ± 2.85 | 63.48 ± 9.63 | 0.006 | 2.08 |

**Table S5.** Monohydroxy lipid metabolite levels (ng/g) in spleen of wildtype and fat-1 mice.

| | Spleen | | | |
|---|---|---|---|---|
| | **Wild Type** | **Fat-1** | ***p*-Value** | **Change (-Fold)** |
| 5-HETE | 2093.74 ± 537.91 | 922.10 ± 236.90 | 0.152 | 0.44 |
| 8-HETE | 1108.33 ± 113.16 | 631.28 ± 31.85 | 0.013 | 0.57 |
| 9-HETE | 3138.36 ± 386.39 | 1820.30 ± 239.05 | 0.040 | 0.58 |
| 11-HETE | 3859.66 ± 287.93 | 2548.80 ± 183.02 | 0.011 | 0.66 |
| 12-HETE | 69,522.40 ± 4007.76 | 49,095.40 ± 1963.00 | 0.005 | 0.71 |
| 15-HETE | 6788.66 ± 1062.22 | 3496.88 ± 355.96 | 0.051 | 0.52 |
| 5-HEPE | 0.00 | 188.03 ± 31.06 | <0.001 | |
| 8-HEPE | 3.23 ± 1.46 | 111.88 ± 28.79 | <0.001 | 34.65 |
| 9-HEPE | 44.34 ± 12.30 | 660.60 ± 219.83 | 0.004 | 14.90 |
| 12-HEPE | 322.46 ± 56.37 | 15,940.73 ± 1875.72 | <0.001 | 49.44 |
| 15-HEPE | 25.39 ± 6.67 | 374.35 ± 53.60 | <0.001 | 14.75 |
| 18-HEPE | 7.79 ± 3.14 | 435.68 ± 99.45 | <0.001 | 55.96 |
| 4-HDHA | 293.79 ± 68.04 | 333.63 ± 66.36 | 0.710 | 1.14 |
| 7-HDHA | 75.10 ± 12.44 | 78.78 ± 18.84 | 0.869 | 1.05 |
| 8-HDHA | 0.00 | 0.00 | | |
| 10-HDHA | 108.81 ± 22.44 | 165.48 ± 27.85 | 0.155 | 1.52 |
| 11-HDHA | 164.00 ± 34.17 | 133.15 ± 24.44 | 0.549 | 0.81 |
| 13-HDHA | 315.16 ± 56.65 | 333.98 ± 70.34 | 0.843 | 1.06 |
| 14-HDHA | 3743.71 ± 486.24 | 4937.35 ± 305.42 | 0.120 | 1.32 |
| 16-HDHA | 137.36 ± 32.51 | 210.53 ± 25.39 | 0.159 | 1.53 |
| 17-HDHA | 867.60 ± 151.58 | 1002.48 ± 137.86 | 0.569 | 1.16 |
| 20-HDHA | 289.57 ± 110.57 | 229.78 ± 83.72 | 0.720 | 0.79 |

**Table S6.** Monohydroxy lipid metabolite levels (ng/g) in heart of wildtype and fat-1 mice.

| | Heart | | | |
|---|---|---|---|---|
| | **Wild Type** | **Fat-1** | ***p*-Value** | **Change (-Fold)** |
| 5-HETE | 1396.27 ± 278.09 | 2040.03 ± 772.40 | 0.364 | 1.46 |
| 8-HETE | 532.61 ± 52.52 | 556.58 ± 167.84 | 0.869 | 1.04 |
| 9-HETE | 545.84 ± 79.97 | 823.48 ± 314.83 | 0.300 | 1.51 |
| 11-HETE | 257.60 ± 33.43 | 522.98 ± 223.20 | 0.148 | 2.03 |
| 12-HETE | 1290.83 ± 140.35 | 1360.05 ± 354.28 | 0.833 | 1.05 |
| 15-HETE | 4070.40 ± 385.24 | 4522.93 ± 1276.74 | 0.680 | 1.11 |
| 5-HEPE | 5.64 ± 1.94 | 112.50 ± 28.89 | <0.001 | 19.94 |
| 8-HEPE | 3.37 ± 0.69 | 47.85 ± 9.32 | <0.001 | 14.19 |
| 9-HEPE | 0.00 | 94.18 ± 89.86 | 0.182 | |
| 12-HEPE | 12.90 ± 5.15 | 137.85 ± 41.00 | 0.003 | 10.69 |
| 15-HEPE | 3.20 ± 1.00 | 60.23 ± 14.65 | <0.001 | 18.82 |
| 18-HEPE | 11.51 ± 5.80 | 433.30 ± 104.04 | <0.001 | 37.63 |
| 4-HDHA | 1259.50 ± 456.93 | 3583.03 ± 1597.38 | 0.110 | 2.84 |
| 7-HDHA | 238.16 ± 29.05 | 766.08 ± 293.73 | 0.037 | 3.22 |
| 8-HDHA | 134.50 ± 13.86 | 589.85 ± 245.66 | 0.031 | 4.39 |
| 10-HDHA | 217.59 ± 23.22 | 783.65 ± 316.13 | 0.037 | 3.60 |
| 11-HDHA | 191.44 ± 22.41 | 645.40 ± 304.49 | 0.072 | 3.37 |
| 13-HDHA | 368.99 ± 47.61 | 1394.90 ± 539.49 | 0.029 | 3.78 |
| 14-HDHA | 729.54 ± 97.13 | 2398.75 ± 852.34 | 0.027 | 3.29 |
| 16-HDHA | 695.97 ± 95.24 | 2623.25 ± 964.76 | 0.024 | 3.77 |
| 17-HDHA | 976.11 ± 131.59 | 3866.85 ± 1415.13 | 0.021 | 3.96 |
| 20-HDHA | 1830.47 ± 272.32 | 6202.93 ± 2246.36 | 0.028 | 3.39 |

**Table S7.** Monohydroxy lipid metabolite levels (ng/g) in skeletal muscle of wildtype and fat-1 mice.

| | Muscle | | | |
|---|---|---|---|---|
| | **Wild Type** | **Fat-1** | ***p*-Value** | **Change (-Fold)** |
| 5-HETE | 250.34 ± 49.48 | 80.55 ± 12.01 | 0.033 | 0.32 |
| 8-HETE | 108.33 ± 26.89 | 46.30 ± 5.13 | 0.124 | 0.43 |
| 9-HETE | 111.09 ± 25.02 | 46.25 ± 4.28 | 0.089 | 0.42 |
| 11-HETE | 109.29 ± 21.70 | 61.48 ± 18.13 | 0.172 | 0.56 |
| 12-HETE | 538.19 ± 35.57 | 283.38 ± 46.99 | 0.002 | 0.53 |
| 15-HETE | 1353.39 ± 140.96 | 539.33 ± 57.89 | 0.002 | 0.40 |
| 5-HEPE | 0.00 | 8.65 ± 3.95 | 0.014 | |
| 8-HEPE | 1.11 ± 0.42 | 5.05 ± 2.93 | 0.106 | 4.53 |
| 9-HEPE | 0.00 | 15.15 ± 8.86 | 0.042 | |
| 12-HEPE | 1.57 ± 0.55 | 39.90 ± 5.30 | <0.001 | 25.39 |
| 15-HEPE | 8.73 ± 2.59 | 22.03 ± 5.37 | 0.032 | 2.52 |
| 18-HEPE | 8.01 ± 2.62 | 66.15 ± 5.79 | <0.001 | 8.25 |
| 4-HDHA | 352.77 ± 128.80 | 234.28 ± 13.47 | 0.515 | 0.66 |
| 7-HDHA | 60.76 ± 11.70 | 86.25 ± 13.26 | 0.202 | 1.42 |
| 8-HDHA | 0.00 | 0.00 | | |
| 10-HDHA | 83.56 ± 12.91 | 94.15 ± 22.17 | 0.666 | 1.13 |
| 11-HDHA | 131.43 ± 8.25 | 166.28 ± 37.19 | 0.262 | 1.27 |
| 13-HDHA | 216.47 ± 35.54 | 261.13 ± 29.88 | 0.419 | 1.21 |
| 14-HDHA | 1062.19 ± 102.00 | 1262.18 ± 183.12 | 0.323 | 1.19 |
| 16-HDHA | 116.83 ± 15.53 | 166.08 ± 27.24 | 0.122 | 1.42 |
| 17-HDHA | 1305.61 ± 147.38 | 1320.80 ± 196.59 | 0.952 | 1.01 |
| 20-HDHA | 429.26 ± 56.80 | 430.08 ± 73.70 | 0.993 | 1.00 |

**Table S8.** Monohydroxy lipid metabolite levels (ng/g) in kidney of wildtype and fat-1 mice.

| | Kidney | | | |
|---|---|---|---|---|
| | **Wild Type** | **Fat-1** | ***p*-Value** | **Change (-Fold)** |
| 5-HETE | 924.41 ± 195.40 | 939.78 ± 366.66 | 0.865 | 1.02 |
| 8-HETE | 305.83 ± 31.80 | 305.93 ± 86.71 | 0.894 | 1.00 |
| 9-HETE | 443.16 ± 41.41 | 364.53 ± 75.13 | 0.459 | 0.82 |
| 11-HETE | 470.84 ± 70.43 | 386.43 ± 148.42 | 0.639 | 0.82 |
| 12-HETE | 1434.39 ± 170.77 | 1247.10 ± 177.96 | 0.662 | 0.87 |
| 15-HETE | 1908.40 ± 270.32 | 2095.10 ± 754.03 | 0.697 | 1.10 |
| 5-HEPE | 1.40 ± 0.92 | 149.63 ± 92.75 | 0.078 | 106.88 |
| 8-HEPE | 1.64 ± 0.51 | 31.85 ± 13.85 | 0.025 | 19.39 |
| 9-HEPE | 13.34 ± 4.03 | 206.90 ± 95.58 | 0.034 | 15.51 |
| 12-HEPE | 12.79 ± 5.41 | 244.10 ± 87.57 | 0.009 | 19.09 |
| 15-HEPE | 5.39 ± 2.41 | 56.98 ± 18.59 | 0.010 | 10.58 |
| 18-HEPE | 5.73 ± 1.52 | 229.70 ± 120.68 | 0.046 | 40.10 |
| 4-HDHA | 772.86 ± 155.78 | 1301.15 ± 627.17 | 0.310 | 1.68 |
| 7-HDHA | 147.66 ± 21.42 | 221.98 ± 72.03 | 0.254 | 1.50 |
| 8-HDHA | 0.00 | 0.00 | | |
| 10-HDHA | 240.07 ± 26.39 | 405.88 ± 133.80 | 0.150 | 1.69 |
| 11-HDHA | 213.20 ± 41.47 | 186.80 ± 29.65 | 0.858 | 0.88 |
| 13-HDHA | 472.06 ± 72.16 | 766.18 ± 236.41 | 0.147 | 1.62 |
| 14-HDHA | 958.07 ± 212.12 | 1593.18 ± 438.81 | 0.075 | 1.66 |
| 16-HDHA | 368.83 ± 43.70 | 477.85 ± 115.12 | 0.200 | 1.30 |
| 17-HDHA | 1276.46 ± 173.59 | 1939.10 ± 464.98 | 0.095 | 1.52 |
| 20-HDHA | 658.74 ± 163.23 | 1004.03 ± 430.90 | 0.305 | 1.52 |
